# Combination of light-driven co-delivery of chemodrugs and plasmonic-induced heat for cancer therapeutics using hybrid protein nanocapsules

**DOI:** 10.1186/s12951-019-0538-3

**Published:** 2019-10-15

**Authors:** E. Villar-Alvarez, A. Cambón, A. Pardo, L. Arellano, A. V. Marcos, B. Pelaz, P. del Pino, A. Bouzas Mosquera, V. X. Mosquera, A. Almodlej, G. Prieto, S. Barbosa, P. Taboada

**Affiliations:** 1 Grupo de Física de Coloides y Polímeros, Departamento de Física de la Materia Condensada, Universidad de Santiago de Compostela, 15782 Santiago de Compostela, Spain; 2 Centro Singular de Investigación en Química Biológica y Materiales Moleculares (CiQUS), Universidad de Santiago de Compostela, 15782 Santiago de Compostela, Spain; 3 Departamento de Cirugía Cardíaca, Complexo Hospitalario Universitario A Coruña, Instituto de Investigación Biomédica de A Coruña (INIBIC), A Coruña, Spain; 4 Department of Physics and Astronomy, College of Science, King Saud University, Riyadh, 11451 Saudi Arabia; 5 Grupo de Biofísica e Interfases, Departamento de Física Aplicada, Universidad de Santiago de Compostela, 15782 Santiago de Compostela, Spain; 6 Instituto de Investigaciones Sanitarias (IDIS) y Agrupación Estratégica de Materiales, Universidad de Santiago de Compostela, 15782 Santiago de Compostela, Spain

**Keywords:** Human serum albumin nanoparticles, Gold nanorods, Multimodal therapy, Stimuli-responsiveness, Photo-therapy

## Abstract

**Background:**

Improving the water solubility of hydrophobic drugs, increasing their accumulation in tumor tissue and allowing their simultaneous action by different pathways are essential issues for a successful chemotherapeutic activity in cancer treatment. Considering potential clinical application in the future, it will be promising to achieve such purposes by developing new biocompatible hybrid nanocarriers with multimodal therapeutic activity.

**Results:**

We designed and characterised a hybrid nanocarrier based on human serum albumin/chitosan nanoparticles (HSA/chitosan NPs) able to encapsulate free docetaxel (DTX) and doxorubicin-modified gold nanorods (DOXO-GNRs) to simultaneously exploit the complementary chemotherapeutic activities of both antineoplasic compounds together with the plasmonic optical properties of the embedded GNRs for plasmonic-based photothermal therapy (PPTT). DOXO was assembled onto GNR surfaces following a layer-by-layer (LbL) coating strategy, which allowed to partially control its release quasi-independently release regarding DTX under the use of near infrared (NIR)-light laser stimulation of GNRs. In vitro cytotoxicity experiments using triple negative breast MDA-MB-231 cancer cells showed that the developed dual drug encapsulation approach produces a strong synergistic toxic effect to tumoral cells compared to the administration of the combined free drugs; additionally, PPTT enhances the cytostatic efficacy allowing cell toxicities close to 90% after a single low irradiation dose and keeping apoptosis as the main cell death mechanism.

**Conclusions:**

This work demonstrates that by means of a rational design, a single hybrid nanoconstruct can simultaneously supply complementary therapeutic strategies to treat tumors and, in particular, metastatic breast cancers with good results making use of its stimuli-responsiveness as well as its inherent physico-chemical properties.

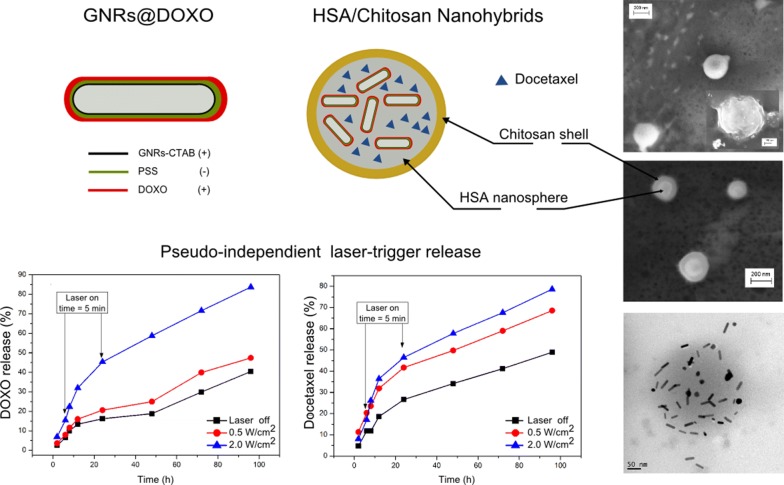

## Background

Breast tumor is the most common type of cancer diagnosed in women and the second one in the number of total deaths worldwide with 252,710 new cases diagnosed in 2017, of which 40,610 led to patient’s death [1]. Doxorubicin (DOXO) is the most widely antineoplasic drug of choice in first-line therapeutic treatments of metastatic breast cancers. Monotherapy with DOXO currently offers good survival rates of ca. 10 to 50% [2], but its applicability is limited due to its inherent associated cardiotoxicity and other additional adverse side effects such as nausea and alopecia after long-term administration [3]. DOXO treatment, as occurred for other single drug cancer therapies, can also fail as a consequence of the development of drug resistances through the generation of inhibitors against apoptotic stimuli or by the activation of multidrug resistant (MDR) genes, which decrease drug uptake and increase its efflux out of malignant cells. This alters the target or metabolic pathway of the antineoplasic compound and activates DNA repair functions in malignant cells [4]. Furthermore, the growth of most tumors is sustained by small populations of “tumor initiating cells” (TICs), which have a high proliferation potential and are inherently resistant, resulting in additional tumor progression and/or recurrence [5].

To overcome these issues, the combined use of different drugs, the so-called combination chemotherapy, has become a key strategy during the last two decades to treat different types of cancer and, in particular, breast tumors [6]. Combination chemotherapy commonly involves the sequential administration of several drugs with different pharmacological mechanisms, and which exert their therapeutic action by means of different synergistic pathways to destroy malignant cells and/or decrease MDR while reduce the associated adverse side effects and related cytotoxicities at the same time [7]. It has been already demonstrated that combination therapy of chemodrugs can provide a synergistic cellular response by acting on multiple pathways, providing an enhanced therapeutic efficacy and reducing the toxicity associated with the administration of higher doses of the individual drugs required to achieve an optimal therapeutic response [8, 9].

Unfortunately, antineoplasic drugs used in conjunction within a single formulation/delivery system are not commercially available so far [10]. The combination of two or more different drugs within one single nanocarrier might help in simplifying tumor treatments, make them much less harmful to patients by allowing: (i) drugs transport to their site of action by passive and/or active mechanisms; (ii) drug protection and avoidance of early clearance from the body; (iii) their sustained and controlled release from the nanocarrier to achieve optimal therapeutic doses in situ; and (iv) the enhancement of their circulation times and the improvement of their pharmacokinetics and pharmacodynamics [11, 12]. To achieve these goals, different types of nanovehicles containing at least two therapeutical compounds with different physicochemical and pharmacological properties have been designed. For example, polymeric-based NPs such as folic acid–modified poly(ethylene glycol)–poly(lactic acid-*co*-glycolic acid) (FA–PEG–PLGA) NPs were used to simultaneously deliver cisplatin (CDDP) and paclitaxel (PTX) for non-small cell lung cancer treatment [13]; poly(ethylene glycol)-block-poly(d,l-lactic acid) (PEG-*b*-PLA) micelles for the co-delivery of PTX and rapamycin (RAP) to endothelial cells (HUVEC) in order to avoid cell proliferation, tubule formation, migration and apoptosis induction [10]; poly(butylene oxide)–poly(ethylene oxide)–poly(butylene oxide) (BO_n_EO_m_BO_n_) block copolymer micelles containing DTX and DOXO to achieve synergistic toxic effects against breast cancer cells [14]; liposomal formulations (some of them already in clinical trials) which combine cytarabine/daunorubicin (CPX-351) and irinotecan/loxuridine (CPX-1) for the treatment of acute myeloid leukemia and colorectal cancer, respectively [15]; chitosan–alginate NPs containing DOXO and vincristine (VCR) encapsulated into vitamin E d-α-tocopheryl polyethylene glycol 1000 succinate-modified PLGA NPs for chemotherapy of lung cancer, Hodgkin’s lymphoma, soft tissue sarcoma, and osteosarcoma [16]; calcium carbonate NPs loaded with drug resistance inhibitors such as celecoxib (CXB) and buthionine sulfoximine (BSO) to downregulate P-glycoprotein (P-gp) expression and depletion of glutathione (GSH) synthesis to inhibit reverse MDR [17]; GNR-based nanocarriers for the co-administration of DOXO and K-Ras targeted small interfering RNA (siRNA) for pancreas cancer therapy [18]; and mesoporous silica NPs (MSNPs) to deliver DOXO and Bcl-2-targeted siRNA to fight against multidrug resistant A2780/AD human ovarian cancer cells [19].

Although proven effective, differences in chemical properties (as molecular weights, solubilities, electrical charges, etc.) and pharmacokinetics of the different bioactive compounds are strong challenges for a successful multidrug loading and transport nanoplatform [20]. In addition, controlled release sequences, rates and profiles of the different encapsulated bioactive compounds are key to achieve optimal and, even, synergistic therapeutic activities. This often requires the design of very complex drug nanocarriers, which are too costly as well as not easily reproducible and scalable for their use in the clinical practice [21].

Single or multidrug chemotherapy can be combined with other therapeutic approaches in order to reduce drug dosages while improving the patients’ survival rates [22]. Particularly, plasmonic photo-thermal therapy (PPTT) is currently under consideration as a non-invasive approach for localized cancer treatment, in which malignant tissues/cells are put in contact with plasmonic NPs and subsequently exposed to light irradiation of suitable wavelength and intensity. This leads to temperature increases exclusively within the tumoral area upon suitable light focalization, providing selective cell killing by means of thermal ablation (T > 45 °C), or providing a thermal sensitization (40 °C < T < 45 °C) to cytotoxic agents: (i) by lowering the hydrostatic pressure of cancerous cells [23] and/or (ii) by increasing the tissue permeability by means of blood vessel dilation, which promotes the uptake of NPs and drugs and, therefore, increases their concentration into malignant cells [24].

Au NPs have been extensively explored as efficient agents for PPTT thanks to their high absorption cross-sections, good biocompatibility, and facile surface functionalization [25]. Among these, GNRs exhibit the most ideal near infrared (NIR) absorption cross-section and demonstrate extremely efficient NIR photothermal heat conversions [26, 27]. GNRs also display tunable longitudinal localized surface plasmon resonances (LPSRs) within the NIR, which is the first optically transparent window of biological tissues (700–1000 nm). Upon NIR irradiation, the excited conduction band electrons of GNRs decay to the ground state by releasing their energy as heat to the surrounding medium [28]. Furthermore, the energy released upon particles irradiation might additionally be used to modify the interactions between adsorbed/attached bioactive compounds on the GNR surfaces, hence, enabling their controlled and sustained release on demand [29].

Hence, in this work we developed a hybrid nanoplatform which enables the simultaneous application of PPTT and combined chemotherapy for metastasic breast cancer treatment by exploiting the photothermal properties of the inorganic part of the hybrid nanocarrier (GNRs) and the ability to co-encapsulate and regulate the release of two different chemodrugs, DOXO and DTX, with complementary cell killing pathways [30]. DOXO can induce DNA damage and apoptosis of cancer cells by inhibiting DNA topoisomerase II, whereas DTX is a cytotoxic agent which inhibits microtubule depolymerization to avoid aberrant mitosis.

**Scheme 1:**
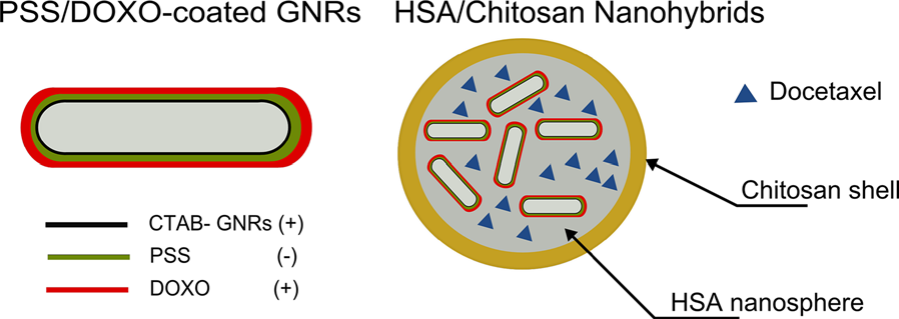
Representation of DTX + PSS/DOXO-coated GNRs@HSA/CS hybrid NPs for combined therapeutics of metastasic breast cancer

The present hybrid nanoplatform consists of human serum albumin (HSA) NPs as carriers able to co-encapsulate the hydrophobic DTX and GNRs (Scheme 1). HSA is the most abundant plasma protein (35–50 g/L human serum), and have shown a preferential uptake in tumor and inflamed tissues, biodegradability, and lack of toxicity and immunogenicity, which make it an ideal building block to construct nanovehicles for controlled drug delivery [31]. In fact, a commercial available platform based on HSA NPs encapsulating the drug DOXO is already approved by the American and European Drug Agencies for the treatment of metastasic breast cancer (ABRAXANE^®^).

GNRs were surface-functionalized through a layer-by-layer (LbL) approach to enable their suitable encapsulation inside the protein nanocarrier as well as to incorporate a second chemodrug, DOXO, on their assembled polymeric surface coating to complement the therapeutic action of loaded DTX inside the protein matrix. To increase the nanoplatform biocompatibility, a chitosan shell was formed around the protein-based nanocarrier taking advantage of the cationic character, good biocompatibility, non-toxicity, biodegradability, mucoadhesivity, and absorption-enhancing effects of this biopolymer [32].

Different amounts of DTX were successfully loaded into PSS/DOXO-coated GNRs encapsulated inside HSA/chitosan NPs (DTX + PSS/DOXO-coated GNRs@HSA/CS NPs). This hybrid nanoplatform exhibited good stability in biologically relevant media. Since DOXO is assembled onto GNRs surfaces and DTX incorporated in the protein matrix, the release rates of both drugs can be regulated pseudo-independently by making use of the sensitivity of the hybrid nanoplatform to the incident NIR light. That is, the latter drug will be mainly released by simple diffusion through the protein carrier whilst the former one is particularly controlled by the localized heat produced on the GNRs surfaces destabilizing their polymeric coating upon NIR light irradiation of the nanoplatform. This hence enables to fit the sequence and extents of released cargoes to the required therapeutic needs.

In addition, the hybrid nanoplatform displayed a good biocompatibility (> 85%), and synergistic cell toxic effects upon dual chemo-treatment were observed after 48 h of incubation. These could be additionally enhanced by exploiting the photothermal properties of GNRs even at low NIR light fluencies within the first 24 h of incubation.

## Materials and methods

### Materials

Hexadecyltrimethylammonium bromide for molecular biology (CTAB), ascorbic acid, tetrachloroauric acid (HAuCl_4_·3H_2_O), silver nitrate (AgNO_3_), sodium borohydride (NaBH_4_), poly(sodium-4-styrenesulfonate) (PSS) of molecular weigh (Mw) ~ 70,000 g/mol, poly-l-lysine hydrobromide (PLL) of Mw ~ 22,000 g/mol, sodium cyanoborohydride (NaCNBH_3_), octyl aldehyde, human serum albumin, lyophilized powder ≥ 97%, and pentasodium tripolyphosphate (TPP) were from Sigma (Sigma-Aldrich Co.; USA). Doxorubicin hydrochloride (DOXO) was purchased from Calbiochem and docetaxel from Acros Organics. Chitosan with an average molecular weight of 415.000 g/mol and 90% degree of deacetylation (Fluka, Cat. no. 28191, middle viscosity grade) was used. All organic solvents were of HPLC grade and all other chemicals were reagent grade commercially available.

### Synthesis of GNRs

GNRs were synthesized using a typical seed-mediated growth method (see Additional file 1 for details).

### Preparation of PSS/DOXO-coated GNRs

Very briefly, for the PSS coating a PSS solution (10 mg/mL) in 12 mM NaCl was prepared. 1 mL of this polyelectrolyte (PE) solution was mixed with 1 mL of a 12 mM NaCl solution, and the resulting mixture stirred at 500 rpm. Then, the latter solution was added dropwise to 1 mL of a GNR solution (OD ~ 1) while stirring. After 1 h of adsorption, the mixture was centrifuged twice at 15,000 rpm for 20 min and redispersed in 1 mL of MilliQ water. Next, a solution of DOXO (1 mM) was prepared in acetic acid buffer at pH 4.1 in order to protonate the amino groups of the drug for a more efficient LbL electrostatic-based deposition. DOXO (typically 100 µg) was diluted in 700 µL of water and stirred at 500 rpm for 5 min. Next, 1 mL of the PSS-coated GNRs was added dropwise to the DOXO solution while stirring. After 1 h, the mixture was centrifuged once at 15,000 rpm for 20 min and redispersed in 1 mL of MilliQ water.

### Preparation of HSA/CS-based NPs

Human serum albumin nanocapsules were prepared by a conventional desolvation method with some modifications, as previously reported [33]. First, a HSA stock solution (20 mg/mL pH 5.5) was made: 5 mg of HSA were mixed with 500 µL of pure ethanol to prepare bare HSA NPs (or with 500 µL of PSS-coated (1·10^11^ NP/mL), or PSS/DOXO-coated GNRs (1·10^11^ NP/mL) as corresponding) at 830 rpm. Then, 0.9 mL of pure ethanol (or 0.9 mL of the desired DTX concentration in ethanol) was added dropwise at a rate of 0.45 mL/min under continuous stirring. Finally, an aliquot of 100 µL of genipin (0.25 mg/mL) was added to cross-link the particles. After the mixture was stirred in the dark for 1 h, the obtained NPs were centrifuged at 15,000 rpm for 20 min and redispersed in 500 µL of MilliQ water. The chitosan shell surrounding the HSA-based NPs was formed using a 10 mg/mL solution of a derivatised chitosan previously obtained [34]. 100 µL of this biopolymer solution were added dropwise to the HSA nanospheres while stirring at 830 rpm. Next, 100 µL of TPP (5 mg/mL) were added to cross-link the chitosan shell. The solution was left under stirring for 1 h and, then, centrifuged at 15,000 rpm for 10 min, followed by resuspension in 500 µL of MilliQ water.

The Au concentration inside the protein NPs was determined by inductively coupled plasma mass spectrometry (ICP-MS) with a Varian 820-MS equipment (Agilent Technologies, USA). To determine the concentration, the Au concentration in 1 mL of GNRs, PSS/DOXO-coated GNRs and PSS/DOXO-coated GNRs@HSA/CS NPs was measured. For bare GNRs, we obtained a concentration of ca. 49.22 ± 0.37 µg/mL (equivalent to 5·10^14^ GNRs/L, rod volume taken as V = [4/3 π R^3^ + πR^2^ (L − 2R)], being L and R the length and the half-width of GNRs measured by TEM). A bulk density of 59 atoms/nm^3^ for metallic Au was considered. PSS/DOXO-coated GNRs and PSS/DOXO-coated GNRs@HSA/CS NPs showed concentrations of ca. 4·10^14^ GNRs/L and 1·10^14^ GNRs/L, respectively. Taking into account these values, it can be estimated that ca. 30% of PSS/DOXO-coated GNRs were encapsulated inside the hybrid protein-based NPs.

### Quantitative analysis of drug loading

To determine the encapsulation efficiency (EE) and loading capacity (LC) of DOXO, PSS/DOXO-coated GNRs were centrifuged at 15,000 rpm at 20 °C for 20 min. The DOXO content in the supernatant was measured by means of UV–Vis and fluorescence spectroscopies using calibration curves previously obtained. These were made using the supernatant of each bare GNRs solution as a blank. The fluorescence standard curve was registered using a λ_exc_ = 480 nm and fluorescence collected at λ_em_ = 560–590 nm. For DTX quantification, HSA/CS NPs were centrifuged at 15,000 rpm at 20 °C for 20 min. The DTX content in the supernatant was measured by means of UV–Vis making use of a previously obtained calibration curve at 230 nm. UV–Vis spectra were measured in a Cary Bio 100 UV–Vis spectrophotometer (Agilent Technologies, USA) whilst fluorescence spectra were monitored in a Cary Eclipse spectrophotometer (Agilent Technologies, USA). Each sample was measured in triplicate for three different batches and averaged. The EE and LC were calculated by the following expressions:

1a $$ EE\left( \% \right) = \frac{Total\;amount\;of\;Drug\;feeded - Drug\;in\;supernatant}{Total\;amount\;of\;Drug\;feeded} \times 100 $$

1b $$ LC\left( \% \right) = \frac{Total\;amount\;of\;Drug\;feeded - Drug\;in\;supernatant}{Total\;weight\;of\;nanoparticles} \times 100 $$

### Dynamic light scattering and ζ-potential measurements

Dynamic light scattering (DLS) and ζ-potential measurements were performed by means of an ALV-5000F (ALV-GmbH, Germany) instrument and a Nano ZS-90 instrument (Nanoseries, Malvern Instruments, UK) (see Additional file 1 for details).

### Transmission electron microscopy and scanning electron microscopy

To acquire transmission electron microscopy (TEM) images, a drop of 5 µL of sample was applied to carbon-coated copper grids, blotted, washed, negatively stained with 2% (w/v) of phosphotungstic acid, air dried, and then examined with a JEOL JEM 1011 (Japan) transmission electron microscope operating at an accelerating voltage of 120 kV. Samples were diluted when necessary before deposition on the grids. To acquire scanning electron microscopy images, a FESEM ultra Plus electronic microscope operating at 20 kV was used. Samples were prepared for analysis by evaporating a drop of the hybrid HSA NP dispersion on a silicon wafer.

### Colloidal stability of protein-based hybrid NPs

The colloidal stability of DTX + PSS/DOXO-coated GNRs@HSA/CS hybrid NPs was assessed by dilution of the samples (1/50) at 37 °C under moderate stirring at 300 rpm for 96 h. ζ-potential and DLS measurements were done using a Zetasizer Nano ZS (Malvern, UK) for several days. The experiments were performed in triplicate for three different batches. The colloidal stability was tested in water and aqueous solutions of pH 5.5 and 7.4 supplemented with 10% (v/v) FBS.

### NIR-laser induced photothermal effect of protein-based hybrid NPs

Temperature increment tests were performed with a continuous wave fiber-coupled diode laser source of 808 nm wavelength (50 W, Oclaro, Inc., San Jose, CA). The laser was powered by a Newport 5700-80 regulated laser diode driver (Newport Corporation, Irvine, CA). A 200-μm-core optical fiber was used to transfer the laser power from the laser unit to the target solution, and was equipped with a lens telescope mounting accessory at the output, which allowed for fine tuning of the laser spot size in the range 1–10 mm. The output power was independently calibrated using an optical power meter (Newport 1916-C), and the laser spot size was previously measured with a laser beam profiler (Newport LBP-1-USB) which was placed at the same distance (8 cm) between the lens telescope output and the cuvette (or the 6-well plate) using the software NewPort LBP series Measurement Systems v3.11. In this way, the power per unit area was easily obtained. The final spot size was set at 1 cm^2^ in diameter. The temperature of GNR samples was measured with a type J thermocouple linked to a digital thermometer inserted into the solutions. Particle solutions were stirred during laser illumination to homogenize the produced heat and ensure that samples were in thermal equilibrium during the entire course of the experiments.

### In vitro release experiments

DOXO and DTX releases from DTX@HSA/CS, PSS/DOXO-coated GNRs@HSA/CS and DTX + PSS/DOXO-coated GNRs@HSA/CS NPs were measured in vitro at a constant temperature of 37 °C under 300 rpm magnetic stirring for 4 days at pH 7.4, and pH 5.5. To obtain the release profiles, 1 mL of protein-based NPs was placed into dialysis tubes (SpectraPore^®^, MWCO 3500) immersed into which 50 mL of buffer supplemented with 10% (v/v) FBS at the pH of interest. The drug concentration released was determined at different time intervals for solution condition. At each sampling time, 1 mL of the medium was withdrawn and replaced with the same volume of fresh buffer to maintain the required sink conditions. The drug content in the supernatant was measured by means of UV–Vis and fluorescence using previously established calibration curves, as described above. Assays were carried out in triplicate. UV–vis spectra were measured in a Cary Bio 100 UV–vis spectrophotometer (Agilent Technologies, USA) for DTX and DOXO. DOXO fluorescence (λ_exc_ = 485 nm; λ_em_ = 580 nm) was measured in a FLUOstar OPTIMA microplate reader (BMG LABTECH, Offenburg, Germany). Assays were carried out in triplicate.

### NIR-light triggered release

In order to analyse the effect of laser exposure in the DOXO and DTX release, 1 mL DTX + PSS/DOXO-coated GNRs@HSA/CS NPs and/or free DTX was incubated at 37 °C under moderate stirring at pH 5.5. The experimental conditions were similar as those previously stated in the absence of light, except that after 6 h and 24 h of incubation hybrid NPs were exposed to NIR light irradiation of 0.5 and 2 W/cm^2^ for 4 min. Experiments were performed with the instrumentation and methodology described above.

### Tumor cells

Cervical HeLa and breast MDA-MB-231 cancer cells from Cell Biolabs (San Diego, CA) were grown at standard culture conditions (5% CO_2_ at 37 °C) in DMEM supplemented with 10% (v/v) FBS, 2 mM l-glutamine, 1% (v/v) penicillin/streptomycin, 1 mM sodium pyruvate, and 0.1 mM MEM nonessential amino acids (NEAA).

### Cellular uptake and DOXO release by fluorescence microscopy

Particle uptake and DOXO release in MDA-MB-231 cells were also followed by fluorescence microscopy. MDA-MB-231 cells were seeded on poly-l-lysine coated glass coverslips (12 × 12 mm^2^, Sigma-Aldrich) placed inside 6-well plates (2 × 10^5^ per well) with 2 mL of DMEM and grown for 24 h at standard culture conditions. Then, hybrid protein-based particles (at a concentration of 1 mg/mL of HSA) were added to cells. After 6 h of incubation, cells were washed three times with PBS and fresh medium was added. Next, some cells were irradiated with a continuous wave fiber-coupled diode laser source of 808 nm wavelength (50 W, Oclaro, Inc., San Jose, CA) for 5 min at a fluency of 0.5 W/cm^2^. After a total desired time of incubation (4, 6, 8, 12, and 24 h), cells were washed three times with PBS, fixed with paraformaldehyde 4% (w/v) for 10 min, washed again with PBS, treated with 0.2% (w/v) Triton X-100 for 10 min and finally washed again with PBS. Then, the coverslips were mounted on glass slides, stained with DAPI (Invitrogen) and cured for 24 h at − 20 °C. Samples were visualized at 63× using a wide field fluorescence inverted microscope (Leica DMI6000B, Leica Microsystems, Germany) using blue channel for DAPI (λ_ex_ = 350 nm), red channel for DOXO (λ_ex_ = 520 nm) and transmitted light in differential interference contrast (DIC) mode. The analysis of fluorescence intensities from the regions of interest (ROIs) of different cells in several microscopic images at the selected time points was done using LAS X Life Science and Image J softwares following a previously established methodology [35].

### In vitro cell cytotoxicity

The cytotoxicity of hybrid HAS-based NPs was tested in vitro by the CCK-8 cytotoxicity assay. MDA-MB-231 cancer cells with an optical confluence of 80–90% were seeded into 96-well plates (100 μL, 1.5·10^4^ cells per well) and grown for 24 h at standard culture conditions in 100 μL growth medium. After 24 h of incubation at 37 °C and 5% CO_2_, 100 μL of several concentrations of NPs (from 4 to 0.25 mg/mL of total HSA) were diluted in the corresponding cell culture medium, injected into the wells and incubated for 6 h. After the corresponding incubation (24 or 48 h), the culture medium was discarded, cells washed with 10 mM PBS pH 7.4, and new fresh culture medium (100 μL) added with 10 μL of CCK-8 reagent to each well. After 2 h, the absorption at 450 nm of the cell samples was measured with an UV–Vis microplate absorbance reader (Bio-Rad model 689). Cell viability was calculated as follows:

2 $$ SR\left( \% \right) = \frac{Abs\;sample}{Abs\;blank} \times 100 $$

where *Abs sample* is the absorbance at 450 nm for samples and *Abs blank* is absorbance for controls without NPs.

In addition, some of the wells were also irradiated with a continuous wave fiber-coupled diode laser source at 808 nm (50 W, Oclaro, Inc., San Jose, CA). The used power fluencies were 0.5, and 2.0 W/cm^2^ for 4 min in each well. After 18 h and 42 h, cells were washed again and new fresh culture medium (100 μL) was added with 10 μL of CCK-8 reagent to each well and measured as specified above.

### Combined indices (CIs)

The combined effect of DTX and DOXO on MDA-MB-231 cells was evaluated for free combined DTX + DOXO drugs solubilized in DMEM or DTX + PSS/DOXO-coated GNRs@HSA/CS NPs. Compusyn software was used for data analysis [36]. This software is based on Chou and Talay’s median effect method [36], in which the median effect equation is a general equation for dose–effect relationship derived from the mass-action law principle that takes into account the potency and the shape of dose–effect curves. The dose–effect relationship as shown by the mass-action law is mathematically described below:

3 $$ \frac{F}{{1 - F_{a} }} = \left( {\frac{D}{{D_{m} }}} \right)^{m} $$

where *F*_*a*_ represents the fraction of cells affected upon drug treatment (1 − SR/100), *D* is the dose of the drug, *D*_*m*_ is the dose required to produce a median effect (e.g., IC_50_), and *m* is the Hill slope (slope of the fit line).

CI values obtained from the software represent the effect of combination. CI value of 1 indicates additive effect; CI > 1 indicates antagonism; and CI < 1 indicates synergism, respectively. CI values of DTX and DOXO were computed using the following formula:

4 $$ CI = \frac{{\left( D \right)_{1} }}{{\left( {D_{x} } \right)_{1} }} + \frac{{\left( D \right)_{2} }}{{\left( {D_{x} } \right)_{2} }} $$

where (*D*_*x*_)_1_ and (*D*_*x*_)_2_ are the inhibitory concentrations of drug 1 and drug 2 alone, respectively. (*D*)_1_ and (*D*)_2_ are the drug 1 and 2 concentrations, respectively. The data were represented as *F*_*a*_-CI plot (Chou-Talalay plot), that is, a plot of CI as a function of effect level (*F*_*a*_).

### Annexin V/dead cell apoptosis assay

MDA-MB-231 cells were treated with bare HSA/CS, PSS-coated GNRs@HSA/CS, PSS/DOXO-coated GNRs@HSA/CS, and DTX + PSS/DOXO-coated GNRs@HSA/CS NPs. Free DOXO, free DTX and free combined DOXO + DTX were used as positive controls. Untreated cells were used as a negative (live) control. After 6 h of incubation with the different formulations, the culture medium was changed by fresh one and cells were illuminated with NIR-light using a CW-808 fiber laser at several power intensities (0, 0.5, 2 W/cm^2^) for 4 min. After 24 h, cells were trypsinized and redispersed in 500 μL of fresh medium (7.5·10^4^ cells/mL). Then, 100 μL of cells were mixed with 100 μL of Annexin V/Dead cell reagent (Muse Annexin V & Dead Cell Assay Kit, Millipore, USA) and incubated for 20 min in the dark at ambient temperature. Finally, flow cytometry was assayed using a Millipore Muse cell analyzer (Millipore).

## Results and discussion

### Synthesis and characterization of the hybrid nanoplatform

HSA NPs were obtained by a desolvation method through the continuous dropwise addition of ethanol to an HSA aqueous solution of pH 5.5 under continuous stirring until the protein solution became turbid. Subsequent crosslinking step with genipin was performed to stabilise the resulting NPs and avoid their disintegration. To construct the hybrid nanoplatform, PSS/DOXO-coated GNRs were encapsulated within the protein NPs during the nanocarrier formation process. PSS/DOXO-coated GNRs were obtained by a seed-mediated methodology following by a layer-by-layer polymeric coating, as previously reported [34] (see “Materials and methods” section and Additional file 1 Figure S1). Free DTX was also incorporated to different extents within the protein NPs when corresponding as mentioned above, to provide the nanoplatform with dual chemotherapeutic activity (see Additional file 1 for further details on DTX loading, and resulting nanocarrier characterization). Finally, an 8-carbon side-chain hydrophobically-modified chitosan previously synthesized in the group [33–38] was adsorbed onto the hybrid HSA NPs to provide the resulting platform with further colloidal stability and cationic electric surface charge. The hydrophobic side chains of the formed chitosan shell may favor larger cell affinities compared to nanocarriers, which only exploit ionic interactions for cell attachment [39]. A schematic representation of the final multimodal hybrid nanoplatform is depicted in Scheme 1.

**Fig. 1:**
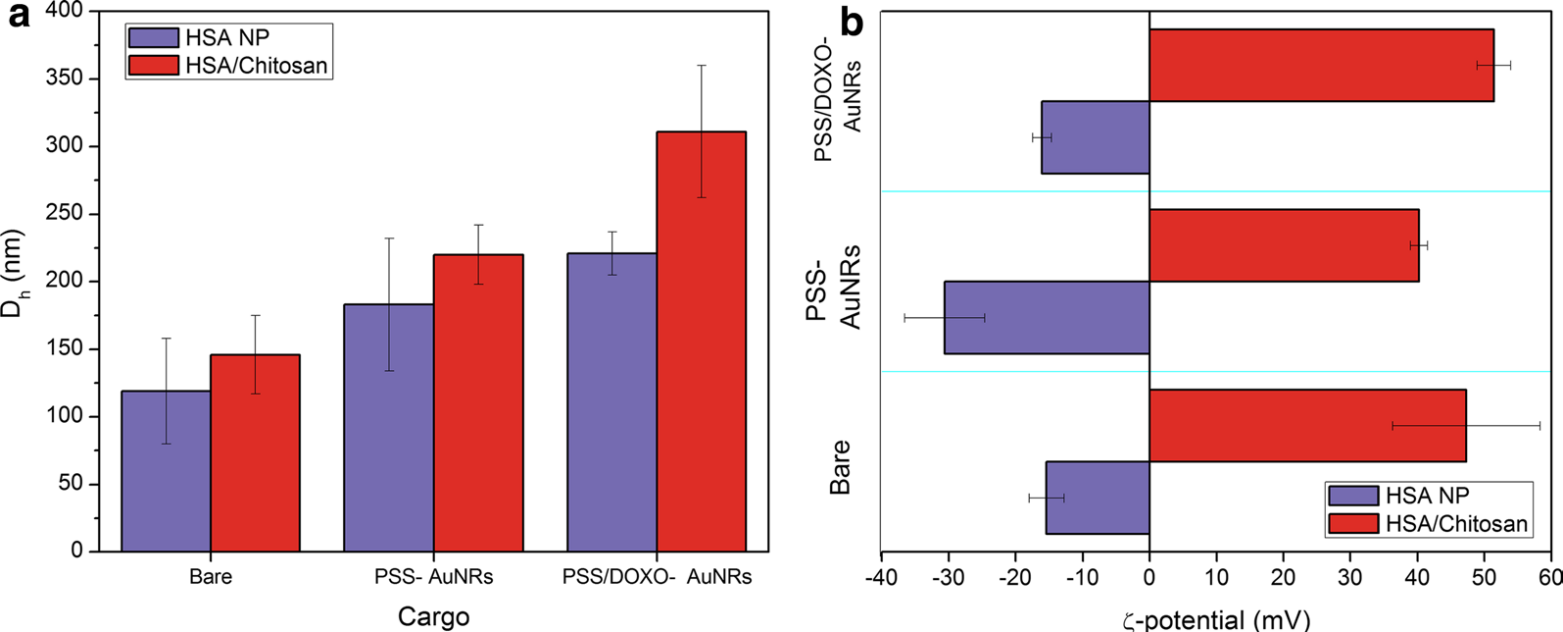
**a** Hydrodynamic diameter (D_h_) obtained from intensity-averaged population distributions, and **b** ζ-potential of HSA (violet) and HSA/CS-based (red) nanohybrids

Bare HSA NPs exhibited sizes of ca. 110 nm, smaller than others previously reported [40, 41] thanks to the modified synthetic procedure followed (Fig. 1a). Hydrodynamic particle sizes slightly increase upon encapsulation of PSS/DOXO-coated GNRs to ca. 210 nm (see Fig. 1) and DTX (see Additional file 1: Figure S3 for further details). Finally, coating the hybrid HSA NPs with the chitosan layer additionally increases the particle sizes between ca. 50–100 nm in diameter.

Bare HSA NPs display a negative surface charge of ca. − 15 mV, which increases to ca. − 30 mV upon the incorporation of PSS-coated GNRs inside the protein particles. When PSS/DOXO-coated GNRs are used instead, ζ-potential of the hybrid protein NPs remains almost constant as a consequence of the screening effect provided by the positively charged amino groups of the drug. The formation of the chitosan shell around the particles completely reverses the particle surface potential, becoming positive (Fig. 1b).

HSA NPs display the typical absorbance peak of proteins at 280 nm. When GNRs are encapsulated inside, a ca. 32 nm-red shift of the LPSR band of the metal NPs is observed compared to free GNRs dispersed in solution. The adsorption of the chitosan shell around the hybrid HSA NPs gives rise to a damping in the LPSR band as a result of the attenuation provided by the denser biopolymeric outer layer (see Additional file 1: Figures S4, S5).

**Fig. 2:**
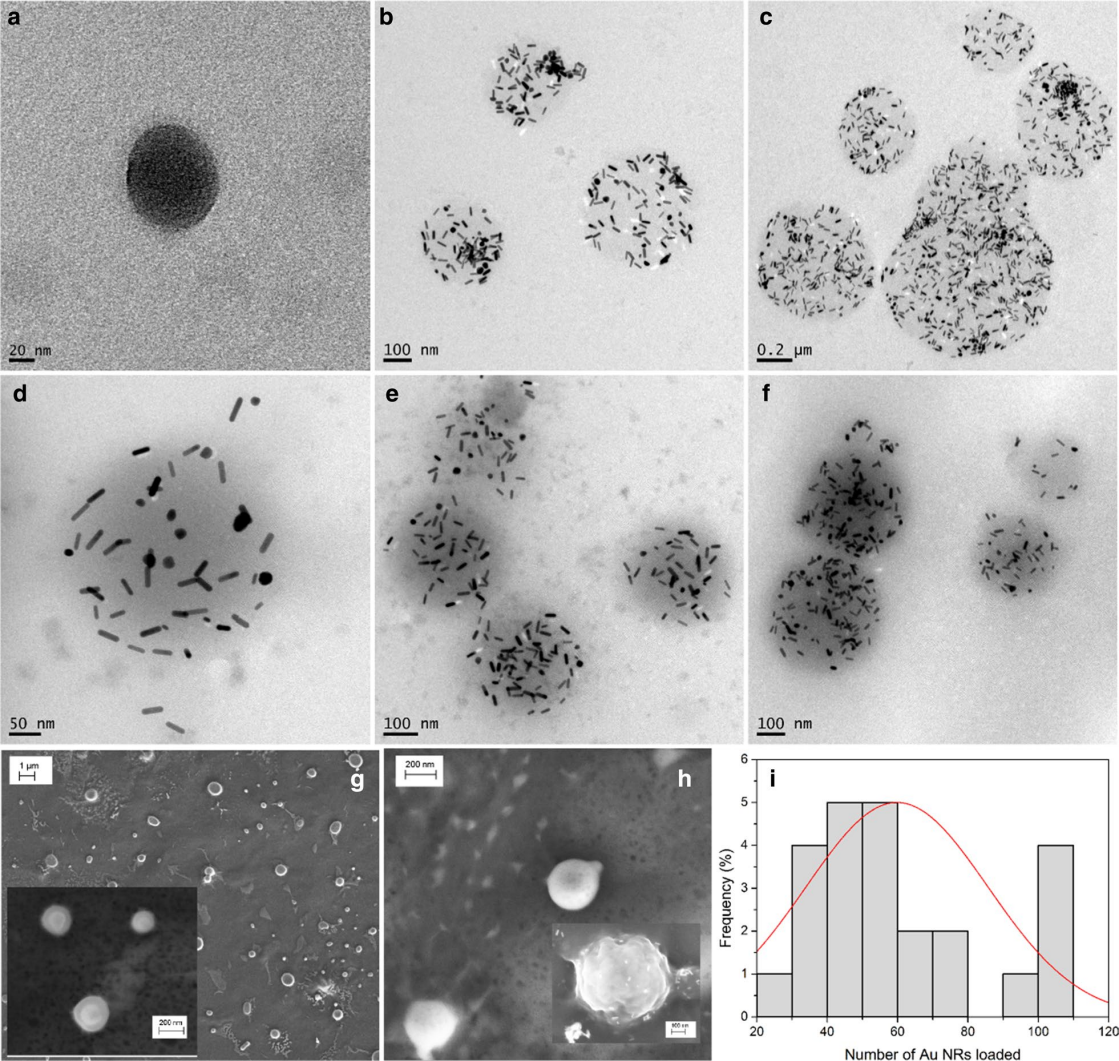
TEM images of **a** bare HSA/CS; **b** PSS-coated GNRs@HSA; **c** PSS/DOXO-coated GNRs@HSA; **d** PSS-coated GNRs@HSA/CS; and **e**, **f** PSS/DOXO-coated GNRs@HSA/CS NPs. SEM images of **g** HSA/CS and **h** PSS/DOXO-coated GNRs@HSA/CS NPs (inset shows PSS/DOXO-coated GNRs@HSA NPs). **i** Frequency distribution of GNRs inside HSA/CS NPs (200 protein particles were analyzed). The Gaussian fit of the distribution is also shown (red line)

Transmission electron (TEM) and scanning electron (SEM) microscopy images of the bare and hybrid protein NPs denote their spherical morphology with sizes ranging between 120 and 310 nm in diameter depending on their composition, in agreement with hydrodynamic sizes (Fig. 2). Bare HSA NPs are observed to be less dense than HSA/CS ones as a consequence of the absence of the chitosan outer layer. Also, these images confirm the successful incorporation of GNRs inside the protein NPs. From inductively-coupled plasma-mass spectrometry (ICP-MS) data we estimated that the extent of GNR incorporation was ca. 30% of the total particle concentration added. We also estimated the loading capacity (LC) and entrapment efficient (EE) of DOXO inside the hybrid protein NPs after PSS/DOXO-GNRs encapsulation to be 0.5 and 74%, respectively whereas that of DTX change from 3 to 5% and 20 to 40%, respectively, depending on the initial fed drug concentration (see Additional file 1 for additional information).

### Colloidal stability of hybrid HSA-based NPs

**Fig. 3:**
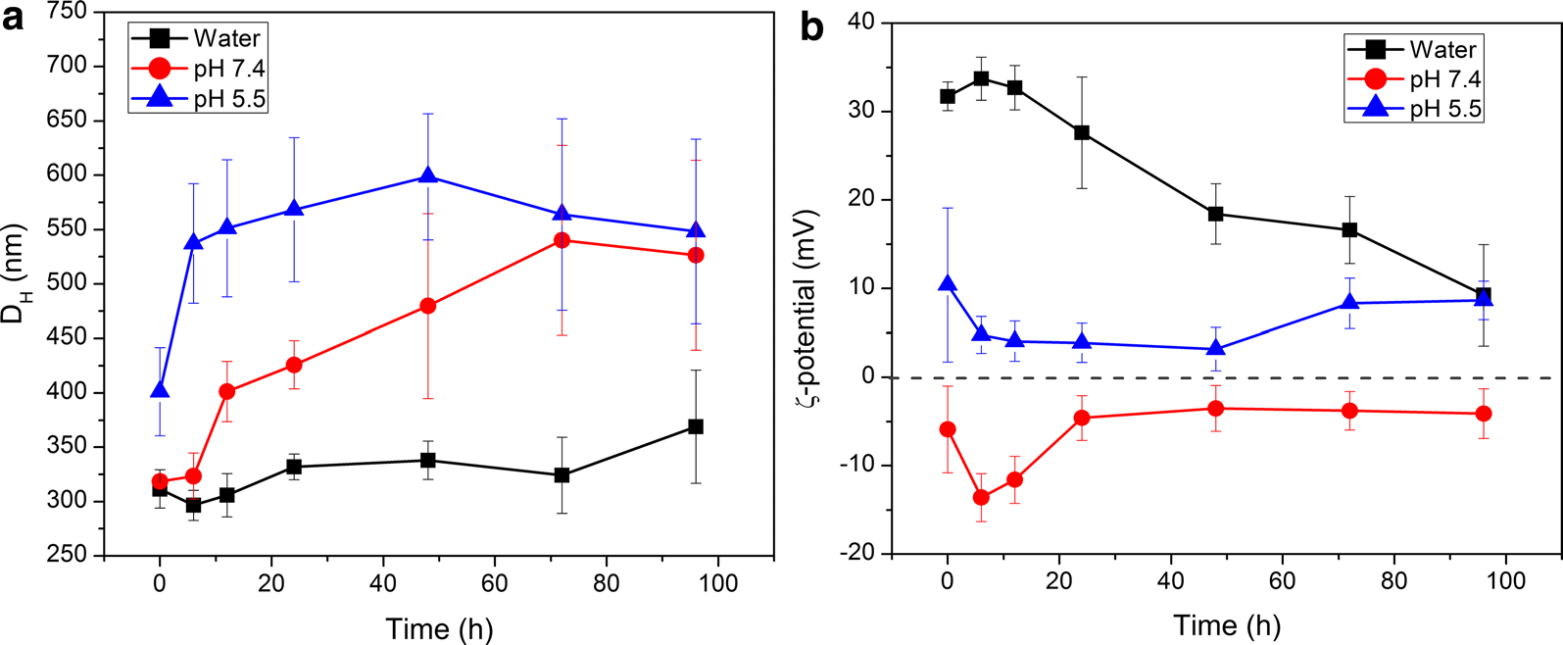
Colloidal stability of DTX + PSS/DOXO-coated GNRs@HSA/CS nanohybrids for 4 days at 37 °C. Samples were dispersed in water (filled black square), and in physiological mimicking media of pH 7.4 (filled red circle) and 5.5 (filled blue triangle) supplemented with 10% (v/v) FBS. Temporal evolution of **a** hydrodynamic particle diameters derived from intensity-averaged size distributions and **b** ζ-potentials of the hybrid HSA-based NPs. Error bars denote the uncertainty of three different measurements

The stability of DTX + PSS/DOXO-coated GNRs@HSA/CS nanohybrids was analysed in pure water and in aqueous solutions of pH of 7.4 and 5.5 supplemented with 10% (v/v) of fetal bovine serum (FBS) to mimic the conditions of blood plasma and the acidic microenvironment of cell cytoplasms (lysosomes), respectively. From Fig. 3a, DTX + PSS/DOXO-coated GNRs@HSA/CS NPs are observed to be stable in water for 3 days and, then, a small particle size increase is noted. At pH 7.4, particle sizes start to grow up after 12 h of incubation as a consequence of the adsorption of proteins onto the shell of the hybrid nanoplatform. This effect is more pronounced at pH 5.5, in which particle sizes are observed to be clearly much larger from the beginning of the incubation process (ca. 400 nm) by means of the formation of a larger protein corona and subsequent particle clustering by means of the interactions between the positively charged NP chitosan shell and the negatively charged proteins present in the aqueous medium.

In addition, ζ-potential values in water of particles are largely positive as corresponds to the outer chitosan layer, and started to decrease after 24 h of incubation likely due to a combination of some potential chitosan desorption and hydrolysis causing scission of the biopolymer chains [42]. At pH 7.4, NPs are negatively charged since chitosan amino groups are neutralized (pK_a_ = 6.5) [33] and carboxylic groups of HSA are ionized, which leads to particle swelling by electrostatic repulsion. The observed ζ-potential decrease at the beginning of the incubation is related to interactions of NPs with surrounding proteins (protein corona formation), which once stabilized allows ζ-potential to be slightly negative and constant. Finally, at pH 5.5 ζ-potentials are lower than in pure water as a consequence of the interactions between serum proteins and the outer chitosan shell, screening the cationic nature of the chitosan shell (Fig. 3b).

### Photothermal properties of hybrid HSA-based NPs

**Fig. 4:**
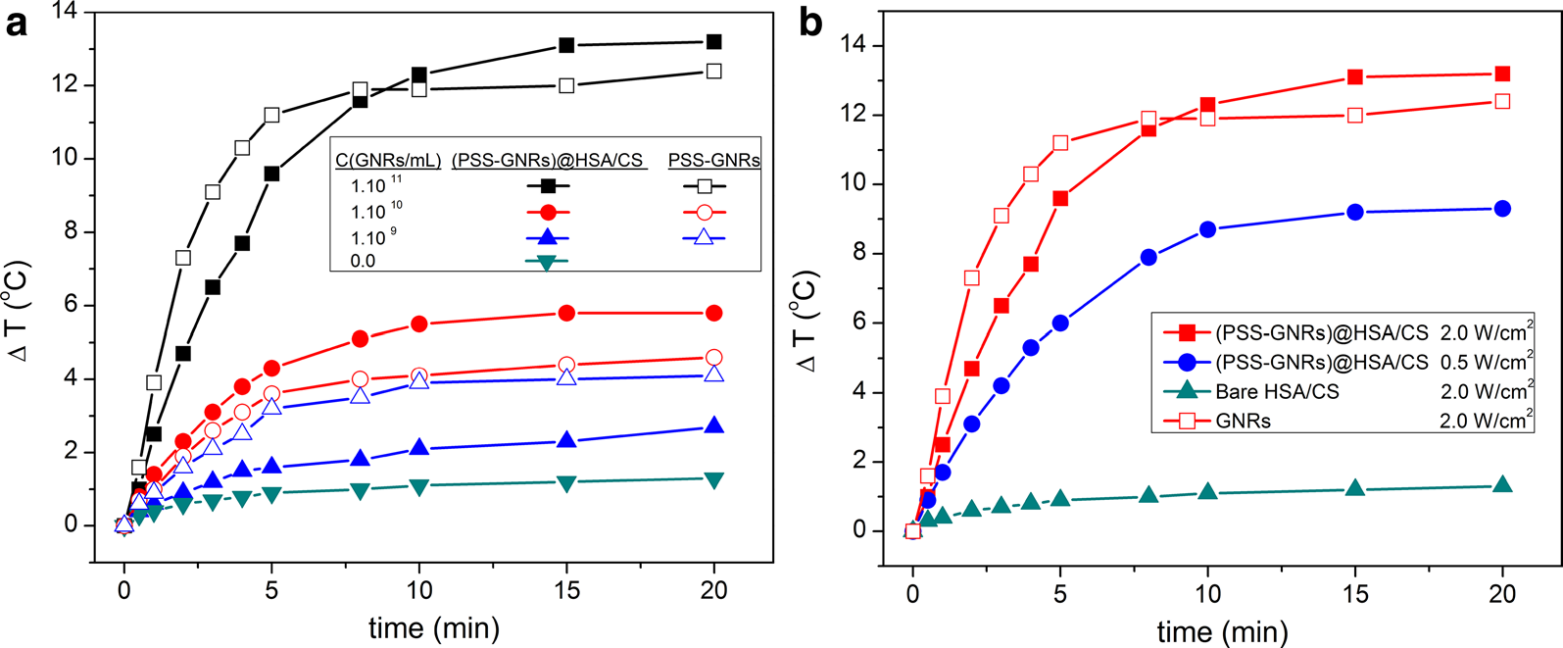
Temperature increases (ΔT) of **a** (PSS-coated GNRs@HSA/CS NPs (close symbols) and non-encapsulated PSS-coated GNRs (open symbols) under NIR light irradiation (808 nm) at a fluency of 2 W/cm^2^ and GNR concentrations 1·10^11^ (filled black square), 1·10^10^ (filled red circle), 1·10^9^ (filled blue triangle) NR/mL and bare HSA/CS NPs (filled green inverted triangle); **b** HSA/CS (PSS-GNRs) NPs irradiated at NIR laser power intensities of 2.0 (filled red square) and 0.5 (filled blue circle) W/cm^2^ ([GNR] = 1.0·10^11^ NP/mL). Bare HSA/chitosan NPs (filled green triangle) and PSS-GNRs (open red square) irradiated at 2.0 W/cm^2^ are also shown as controls. Error bars are not shown for clarity. Uncertainties are within ± 10%

Some chemotherapeutic agents give rise to more important cytotoxic effects at elevated temperatures. However, the underlying mechanism of such heat-induced enhancement is not completely understood [43]. The combination of chemo-drugs with applied focused heating can help to diminish the drug dosage requirements to achieve localized, comparable (and even larger) cytotoxic effects while reducing undesirable side reactions [44]. Hence, temperature increments of PSS-coated GNRs@HSA/CS NPs in aqueous solution of pH 5.5 under constant 808 nm NIR light irradiation conditions were recorded at different particle concentrations (Fig. 4a) and at different power densities (0.5 and 2.0 W/cm^2^) (Fig. 4b). Similar concentrations of free PSS-coated GNRs/mL were used as reference controls.

Negligible temperature increments are induced upon illumination of bare HSA/CS NPs (maximum of ca. 1 °C at 2 W/cm^2^ after 20 min of irradiation, see Fig. 4a). For PSS-coated GNRs@HSA/CS NPs heating profiles denote temperature increments of up to ca. 13, 5 and 2 °C after irradiation for 10 min at GNR concentrations of 1·10^11^, 1·10^10^ and 1·10^9^ GNR/mL, respectively. Surprisingly, these increments are larger than those observed for free GNRs in solution at similar concentrations. This observation might be accounted for the different environments experienced by encapsulated and free GNRs, with different dielectric and heat transfer properties, as well as certain enhanced excitation due to plasmon coupling in the former [45]. As a result, the confinement of GNRs into HSA/CS NPs results in relatively faster and larger temperature increases of the surrounding aqueous medium. In addition, the shape of the temperature profiles (a constant heating of the particle solution upon initial irradiation until reaching a thermodynamic equilibrium) is quite similar to other previously reported NIR-sensitive nanomaterials as other metal nanoparticles such as, gold nanostars [46, 47], gold nanoshells [48], gold nanocages [49], gold nanoprisms [50]; gold-hybrid nanoparticles, capsules and liposomes, [51–53], iron oxide and copper-sulfide nanoparticles [54, 55], carbon-based materials such as carbon nanotubes and graphene oxide [56, 57], and NIR-active fluorophore-loaded particles [24, 58, 59].

The observed temperature increments in solution are dependent mostly on the particle concentration and irradiation conditions; at this respect, but the obtained heat response of our hybrid protein-based nanoparticles lied within rather similar values to those obtained for other gold-NP encapsulated hybrid particles and capsules under similar irradiation conditions using a CW NIR-laser and metal content [51–53, 59]. Moreover, temperature increments for most of particle concentrations and fluencies tested are within the optimal window to allow the use of these hybrid NPs as photothermal agents for localized hyperthermia therapy in order to induce cell apoptosis (between 42 and 47 °C, ΔT = 5–10 °C). For example, at a fixed GNR concentration under irradiations of 2 and 0.5 W/cm^2^ the maximum temperature increments are reached within 4 and 8 min, respectively (Fig. 4b), but always within the limits of the maximum permissible time exposure (MPE) and intensity (ca. 3.3 W/cm^2^ for an CW 808 nm laser) [60, 61].

### Drug release from hybrid HSA-based NPs

The cumulative DOXO and DTX release profiles from DTX + PSS/DOXO coated-GNRs@HSA/CS NPs at neutral and acidic conditions supplemented with 10% (v/v) FBS in the absence and presence of NIR light irradiation of 0.5 and 2.0 W/cm^2^ were obtained. DTX@HSA/CS and PSS/DOXO-coated GNRs@HSA/CS NPs were analysed for comparison.

In the absence of NIR illumination, release profiles for both DTX and DOXO at the different solution conditions display an initial burst phase followed by a sustained release pattern. In general, the initial fast release takes place within ca. first 10 h, with DTX and DOXO releases lying between ca. 10–30% and 13–18%, respectively, depending on the solution pH and the composition of the nanocarrier. This initial leakage from the particles is rather lower than that observed from different polymeric nanoparticles and micelles [62–64], nanogels [65] or solid lipid nanoparticles [66], and similar to that of many other anticancer drug-liposome [67, 68] and polymeric micelles and particles-based [14, 69, 70] formulations. However, it is worth recognising that nowadays some few different nanocarriers have designed that completely block the uncontrolled premature leakage of the cargo and allowing its complete release in the targeted site on-demand under controlled internal or external stimuli [24, 35, 71, 72].

**Fig. 5:**
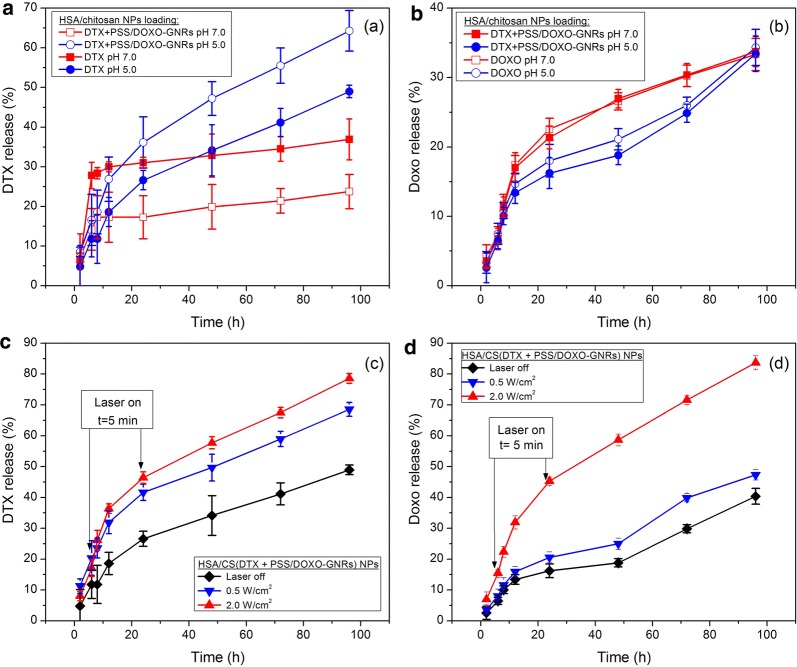
**a** DTX and **b** DOXO cumulative releases from DTX@HSA/CS (open symbols) and DTX + PSS/DOXO coated-GNRs@HSA/CS NPs (closed symbols) at pH 7.4 (filled red square, open red square) and pH 5.5 (filled blue circle, open blue circle). NIR-induced releases of **c** DTX and **d** DOXO from DTX + PSS/DOXO coated-GNRs@HSA/CS NPs at pH 5.5 at 2.0 (filled red triangle), 0.5 (filled blue inverted triangle) and 0 (filled black diamond) W/cm^2^. Lines are only to guide the eye

In addition, DTX is released faster and to larger extents (ca. 65%) from bare DTX@HSA/CS NPs under acidic conditions as a consequence of the protonation of the chitosan shell (Fig. 5a), which leads to a certain collapse of the NP, as reported elsewhere [38]. Strikingly, when DTX is encapsulated within PSS/DOXO-coated GNRs@HSA/CS NPs, the amount of DTX released at short incubation times at pH 7.4 is larger than at pH 5.5 (30% compared to ca. 18% at 10 h); nevertheless, at longer incubation times the cumulative release at the latter pH becomes much greater (ca. 50% compared to 35% at pH 5.5 and 7.4, respectively). Changes in ionization states of DOXO, the chitosan shell and HSA residues would give rise to changes in the extent of swelling of the hybrid NPs, thus, affecting the release profiles. For DOXO, cumulative releases seem to be slightly larger at pH 7.4 than at 5.5 but with low released amounts, ca. 30–35% after 96 h of incubation. The presence of DTX does not have any influence on the DOXO cumulative release (Fig. 5b).

On the other hand, DTX and DOXO releases were enhanced under NIR light irradiation at 808 nm (Fig. 5c, d), in agreement with other NIR-sensitive drug delivery systems [24, 71, 72]. In particular, 67 and 78% of DTX were released after 96 h of incubation under 0.5 and 2.0 W/cm^2^ NIR light irradiation at pH 5.5, much larger than in the absence of irradiation (ca. 50%). As DTX is simply dispersed inside the HSA/CS matrix, the observed differences in cumulative releases of this drug would correspond to an enhanced swelling of the protein particle core and/or higher drug diffusion through the protein matrix as a consequence of the temperature increment under NIR light irradiation.

DOXO release from the hybrid particles in the presence of NIR light also greatly increases; this enhancement is particularly very large at the highest fluency used (2 W/cm^2^, ca. 85% of cargo released). In this case, higher fluencies can allow enhanced DOXO detachments from GNR surfaces by modulating the underlying electrostatic interactions between the drug and the PSS polyelectrolyte layer [73]. Hence, by modulating the NIR fluency it is then possible to tune the release ratio between DTX and DOXO looking for maximizing the therapeutic outcome of the nanoplatform by exploiting the different mechanisms of action of both drugs, and minimizing the required total chemical therapeutic dose.

### Cell uptake and intracellular distribution of hybrid HSA-based NPs

The in vitro uptake and cellular distribution of hybrid HSA-based NPs, particularly PSS/DOXO-coated GNRs@HSA/CS NPs, in breast MDA-MB-231 cancer cells were qualitatively analysed by fluorescence microscopy taking advantage of the red fluorescent properties of DOXO [74]. DOXO release from the hybrid particles was tracked for 24 h at several time intervals and compare to that of free DOXO uptake (positive control), and PSS-coated GNRs@HSA/CS NPs (negative control). Besides, the influence of low-power NIR light irradiation (0.5 W/cm^2^) was depicted too.

**Fig. 6:**
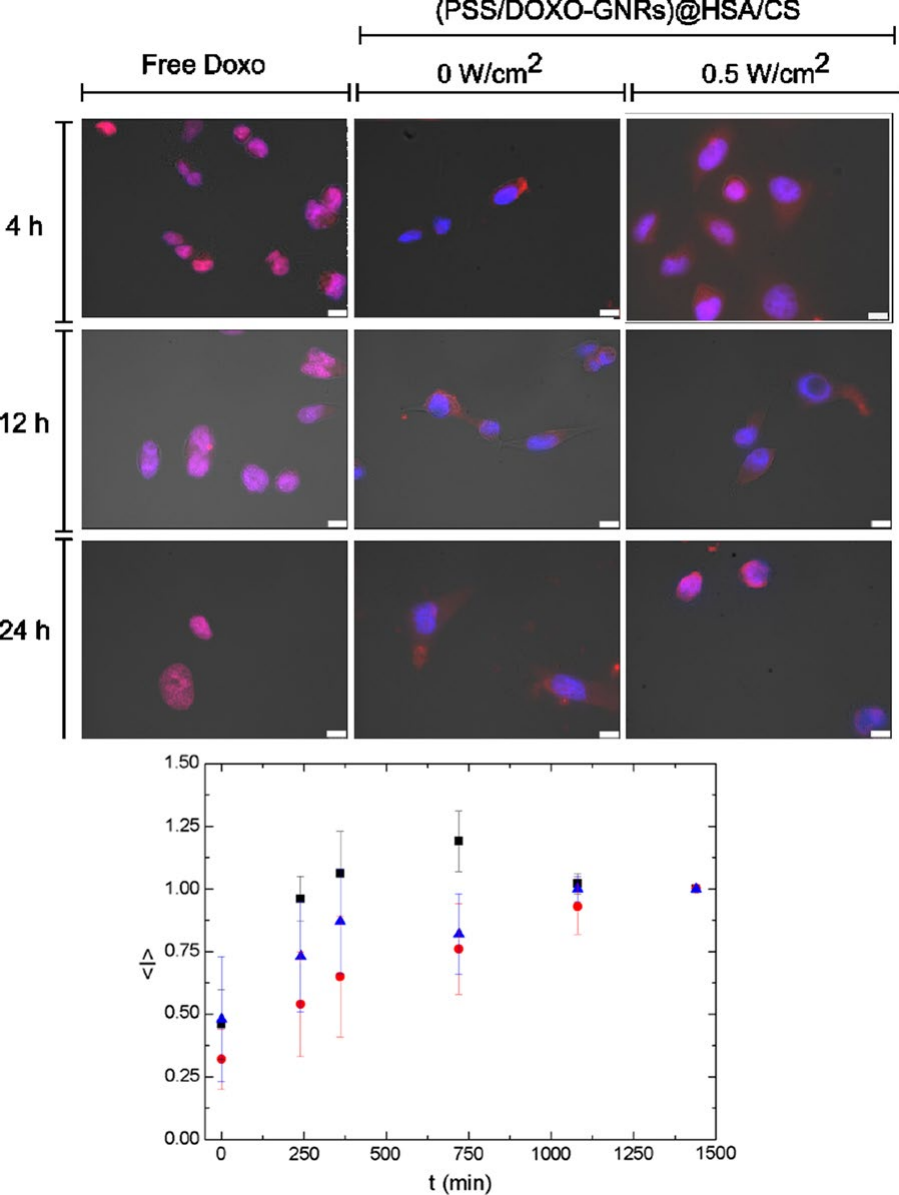
Fluorescence microscopy images of particle cellular uptake and subsequent intracellular DOXO release in breast MDA-MB-231 cancer cells. Free DOXO and (PSS/DOXO-coated GNRs@HSA/CS NPs are shown at different time points in the presence and absence of NIR light illumination (0.5 W/cm^2^ at 808 nm for 5 min). Red channel was from DOXO fluorescence (λ_exc_ = 488 nm); blue channel from cell nuclei stained with DAPI (λ_exc_ = 355 nm); bright field channel and merged images are also shown. Scale bar is 10 µm. The plot represents the average normalized measured intensity values (in the region if interest, nuclei) of doxorubicin per cell (in the region of interest, nuclei, of several cells). The intensity was made in relation to the fluorescent intensity signal at the longest incubation time (24 h)

Figure 6 and Additional file 1: Figure S6 confirmed that DOXO encapsulated within the hybrid NPs is effectively internalized into tumor cells and start to diffuse along the cell cytoplasm, at least, after 4 h (see also 3D reconstructed images in Additional file 1: Figure S7). Conversely, free DOXO rapidly diffuses into cells through the membranes and passes to the nuclei before 4 h of incubation, as confirmed by the pink-colored nuclei in the merged images (Fig. 6). In the absence of light irradiation, a certain sustained release of DOXO from PSS/DOXO-coated GNRs@HSA/CS NPs can be envisaged thanks to the progressive spread of red and pink colours along the cell cytoplasms as the incubation time increases. In the presence of NIR light irradiation, a faster diffusion of the drug can be observed, that is, a relatively more intense red and pink colour scheme is present at shorter incubation times (Fig. 6). On other hand, bare PSS-coated GNRs@HSA/CS NPs only exhibited some bright red points corresponding to light reflectance from the metal NPs (see Additional file 1: Figures S4, S5).

### In vitro cytotoxicity of hybrid HSA-based NPs

The potential cytotoxicity of bare HSA/CS and PSS-coated GNRs@HSA/CS NPs at different particle concentrations was evaluated by means of the CCK-8 proliferation assay in a breast MDA-MB-231 cancer cell line in vitro at different time points (24 and 48 h of incubation). Both types of particles display a concentration-dependent toxicity, with cell viabilities above 50% except for concentrations above 2 mg/mL after 48 h of incubation (see Additional file 1: Figure S8). No significant differences in cell toxicity were observed in the absence and presence of encapsulated PSS-coated GNRs inside the HSA NPs. In the light of these results, a NP concentration of 1 mg/mL was used in subsequent experiments.

### Chemotherapeutic effect of hybrid HSA-based NPs

**Fig. 7:**
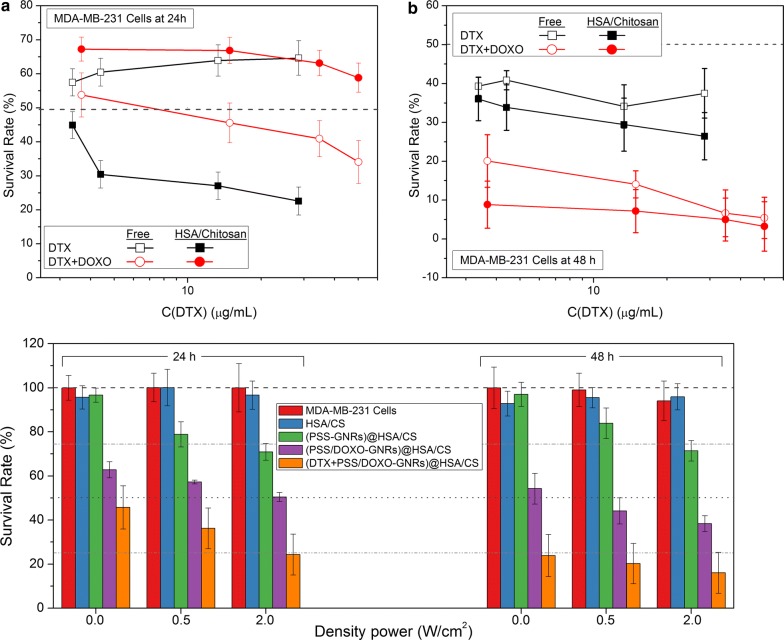
Cellular viability of drug-loaded GNRs@HSA/CS hybrid NPs (closed symbols), and free drugs (open symbols) expressed as survival rates for free DTX (filled black square, open black square) and free DTX + DOXO (filled red circle, open red circle), respectively, in breast MDA-MB-231 cancerous cells for **a** 24 h and **b** 48 h of incubation as a function of DTX concentration. **c** Cell viability induced by 808 nm CW laser illumination at several power intensities (0.0, 0.5, 2.0 W/cm^2^) after 24 and 48 h of incubation. Bare cells (red) and cells treated with bare HSA/CS (blue), PSS-coated GNRs@HSA/CS (green), PSS/DOXO-coated GNRs@HSA/CS (violet), DTX + PSS/DOXO-coated GNRs@HSA/CS NPs (orange) were irradiated for 4 min after 6 h of incubation in MDA-MB-231 cells. Dashed (survival rate at 100%), dotted (survival rate at 50%), dashed dot (survival rate at 75%) and dashed-dot-dot (survival rate of 25%) lines are only to guide the eye

Next, the cytotoxicity of free DTX, free DTX + DOXO, DTX@HSA/CS and DTX + PSS/DOXO-coated GNRs@HSA/CS NPs was also assessed using the CCK-8 assay (Fig. 7). Free DOXO and PSS-coated GNRs@HSA/CS NPs were also assayed as controls (see Additional file 1: Figure S9). A constant DOXO concentration was considered (43.5 µg/mL), whereas the DTX concentration was varied in the range from 3 to 50 µg/mL. Encapsulation of DOXO inside the hybrid NPs led to a cell cytotoxicity of ca. 50% after 48 h of incubation, slightly lower than free administered DOXO (Additional file 1: Figure S7). This is a consequence of the sustained release of the encapsulated drug from the hybrid nanoplatform, as observed in Figs. 5b and 6 and in other nanocarriers [24, 46, 47, 69]. In the case of DTX, the observed cell viabilities for free DTX are concentration-independent and close to 60% and 40% after 24 and 48 h of incubation, respectively; in contrast, cell toxicities were larger for encapsulated DTX inside the NPs, showing a slight concentration-dependent profile with survival rates ranging from 45 to 25%. Finally, when DOXO and DTX are combined within the hybrid nanocarrier, the observed survival rates ranged between 60 and 65% after 24 h of incubation, larger than that of the free combined DTX + DOXO (ranging from 55 to 35% as the total drug concentration increases). This difference can be a consequence of the sustained release pattern of both encapsulated drugs. This is confirmed under longer incubation (48 h), at which cell toxicity largely increases up to ca. 90–95% for DTX + PSS/DOXO-coated GNRs@HSA/CS hybrid NPs thanks to sustained release of the drugs from the platform, and is much higher than that of free DTX + DOXO concentrations (ca. 65–70%). This larger toxic effect has been attributed to the targeting delivery of the nanocarrier to the cancerous cells and the synergistic effect provided by the released of the co-encapsulated drugs, as observed for some other formulations simultaneously encapsulating several chemodrugs as liposomes [68, 74], polymeric micelles and nanoparticles [8, 9, 14, 16], nanogels and hydrogels [75, 76], inorganic nanoparticles [18, 19], etc.

In summary, very large cell mortalities from the combined DTX + DOXO encapsulated drugs were achieved (ca. 90%) by only using 20–30% of the total amount of the free combined drugs concentration in solution required to attain similar cell mortalities. It is also of great importance the sustained release of the drugs from the hybrid nanoplatform-thus overcoming one of the main drawbacks of free drug(s) administration, this is, their short circulation half-lives (ca. 13 h) [77].

### Combination index (CI) analysis

To analyse whether encapsulated DTX and DOXO combination therapy within the hybrid HSA-based NPs is synergistic, additive, or antagonistic against MDA-MB-231 proliferation, combination indices (CIs) for different DTX/DOXO dose ratios were calculated using Compusyn software [36] (see further details in Additional file 1). CIs for the hybrid nanoplatform encapsulating both DOXO and DTX are shown in Additional file 1: Figure S10 and Table S1. Additional file 1: Figure S10 shows that for the combined dual drug therapy at 24 h, and especially for free drug administration, a strong antagonistic effect is noted. Conversely, after 48 h a strong synergistic effect was confirmed, particularly for DTX + PSS/DOXOcoated-GNRs@HSA/CS hybrid NPs. It is also worth mentioning that the total drug concentration released from the NPs is lower than that of free administered antineoplasics, so that the therapeutic effect results more sustained in time and should avoid adverse side effects related to excessive drug concentrations in plasma, as mentioned previously.

IC_50_ values were obtained from the former analysis, being in fair agreement with those previously reported (IC_50_ (DTX) ~ 0.0572 µM and IC_50_ (DOXO) ~ 1.24 µM) for MDA-MB-231 cells after 48 h of incubation. It can be observed in Table 1 that for DTX + PSS/DOXO coated-GNRs@HSA/CS hybrid NPs the individual dose required to exhibit a 50% of cytotoxicity is smaller than doses required for free chemodrugs, then highlighting the effectiveness of the hybrid NPs as suitable nanocarriers.

**Table 1:** IC_50_ values for free and encapsulated DOXO and DTX inside HSA/CS NPs in MDA-MB-231 breast cancer cells by means of Compusyn software

	DOXO (µM)		DTX (µM)	
	Free	Loaded	Free	Loaded
24 h	6.99 ± 0.75	2.52 ± 0.42	0.22 ± 0.07	0.38 ± 0.09
48 h	1.41 ± 0.21	0.99 ± 0.15	0.11 ± 0.04	0.09 ± 0.04

### Photothermal effect of hybrid HSA-based NPs

To analyse the potential combination of the photothermal capabilities of the hybrid platform with the chemotherapeutic effect provided by encapsulated DTX and DOXO, additional cytotoxicity experiments were performed for DTX + PSS/DOXO coated-GNRs@HSA/CS hybrid NPs by means of the CCK-8 assay in the presence of NIR light illumination (808 nm) at different intensities (0, 0.5 and 2 W/cm^2^) for 4 min (see “Materials and methods” section for details). Breast MDA-MB-231 cancer cells were treated with free DOXO, free DTX and free DTX + DOXO (DTX/DOXO ratio of 6.5), bare HSA/CS, PSS-coated GNRs@HSA/CS, PSS/DOXO-coated GNRs@HSA/CS and DTX + PSS/DOXO-coated GNRs@HSA/CS hybrid NPs. The hybrid NP concentration was fixed at 1 mg/mL of HSA (i.e., 1·10^10^ GNRs/mL).

It was observed that both blank cells and those treated with HSA/CS NPs showed viabilities above 95% under NIR light irradiation corroborating its harmless effect (Fig. 7c). In comparison, NIR laser illumination of cells treated with PSS-coated GNRs@HSA/CS particles led to a certain noticeable increase in toxicity as a result of the previously observed temperature increments (see Fig. 4) provided by the hybrid NPs. In particular, cell mortality at 0.5 W/cm^2^ was ca. 22 and 15% at 24 and 48 h of incubation, respectively, and at 2 W/cm^2^ this only increased by an additional 10% after 48 h of incubation, similar to that observed for other previously reported gold nanorod-based nanoplatforms as photothermal agents [27, 29, 30].

Surprisingly, for PSS/DOXO-coated GNRs@HSA/CS NPs a drastic decrease in cell viabilities to ca. 60 and 50% and 50 and 40% at 0.5 W/cm^2^ and 2 W/cm^2^ after 24 and 48 h of incubation, respectively, is observed. However, such mortality mainly stems from the presence of DOXO in the hybrid particles, as observed from the corresponding control experiments. Hence, in order to enhance the effect of photothermal therapy, larger fluencies and/or exposure times and/or larger concentrations of encapsulated GNRs might be needed.

Finally, when both DOXO and DTX are combined within DTX + PSS/DOXO-coated GNRs@HSA/CS hybrid NPs cell death largely increases, especially after 48 h of incubation. Laser exposure, in this case, helps to reduce cell survival rates compared to non-irradiated samples up to ca. 20 and 16% at 2 W/cm^2^ after 24 and 48 h of incubation, respectively. More than a pure hyperthermic effect, the laser illumination may provide thermal sensitization promoting the uptake of particles by increasing cell permeability and subsequent drug release inside cytoplasm [77].

### Cellular death

To determine the main mechanism by which the present multifunctional hybrid nanoplatforms cause toxicity to cells, an Annexin V/Dead cell assay was performed. MDA-MB-231 cells were administered with bare HSA/CS, PSS-coated GNRs@HSA/CS, PSS/DOXO-coated GNRs@HSA/CS and DTX + PSS/DOXO-coated GNRs@HSA/CS hybrid NPs. Untreated cells were used as a negative control whereas free DOXO, free DTX and free DOXO + DTX were used as positive ones. After 6 h of incubation, cells were subjected to NIR light illumination of different fluencies (0, 0.5 and 2.0 W/cm^2^) for 4 min. The proportion of live, dead and apoptotic cells was quantified by flow cytometry.

**Fig. 8:**
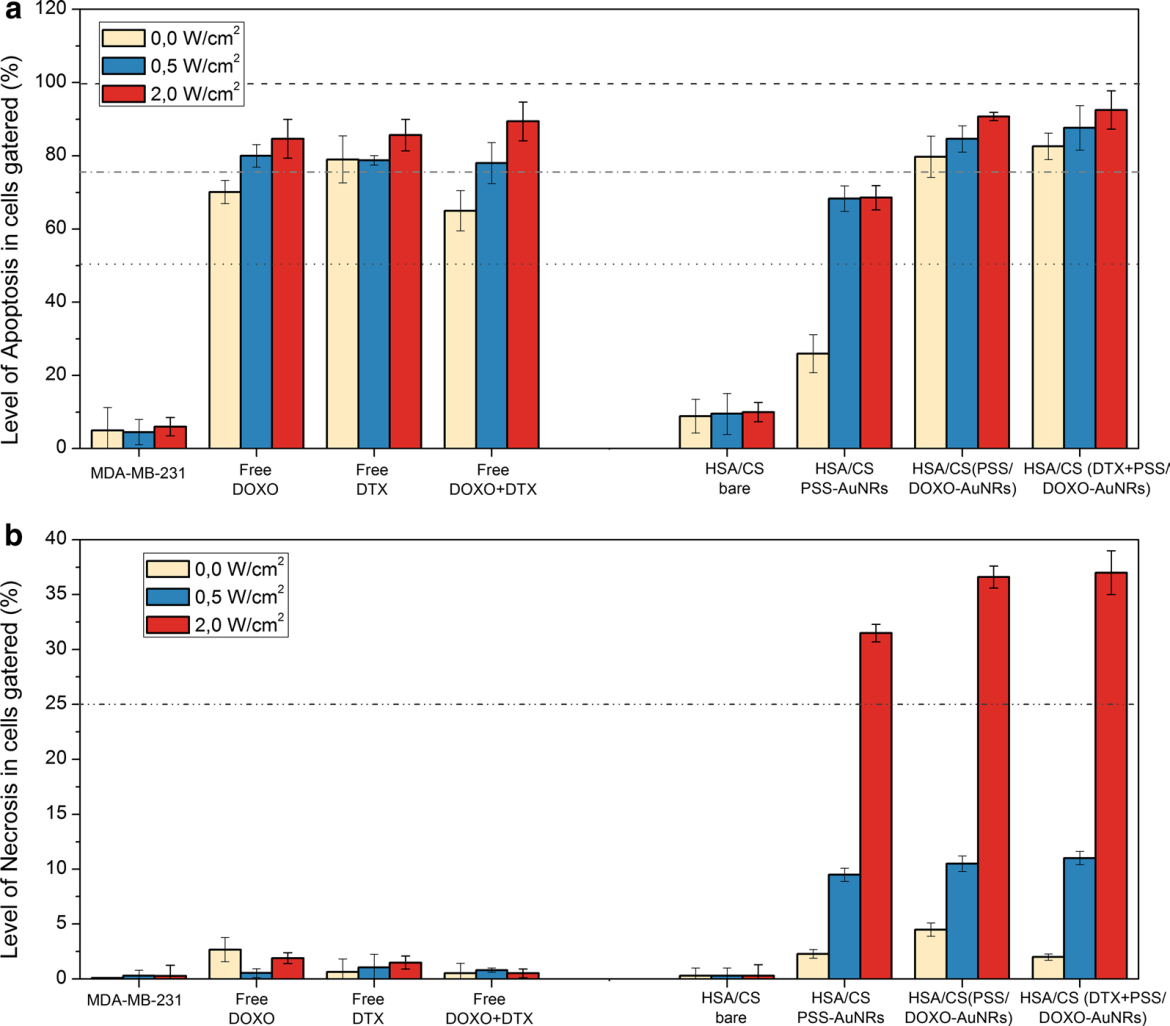
Annexin V/Death cell assay in MDA-MB-231 cells in **a** apoptosis and **b** necrosis. Bare HSA/CS, PSS-coated GNRs@HSA/CS, PSS/DOXO-coated GNRs@HSA/CS and DTX + PSS/DOXO-coated GNRs@HSA/CS hybrid NPs were irradiated at several power densities with a NIR 808 nm CW laser at 0.0 (white), 0.5 (blue) an 2.0 W/cm^2^ (red). Free DOXO, free DTX and free DOXO + DTX were used as positive controls, whereas untreated cells were the negative one

Figure 8 shows that untreated cells display small fluorescent signals for both Annexin V and 7-ADD, as expected. Free single DOXO and DTX-treated cells exhibit a level of apoptosis ranging from 65 to 79% in both cases. Furthermore, for combined free DOXO + DTX, but a notable increment in apoptosis with light fluency is also observed. A slight signal from 7-ADD is also present, but this is below 5% and which can be associated to secondary necrosis, that is, the loss of membrane integrity by late apoptotic cells [52]. Bare HSA/CS NPs display apoptotic levels of ca. 8% without an appreciable role played by light irradiation. Experimental data confirmed that cell death caused by hybrid NPs, PSS/DOXO-coated GNRs@HSA/CS and DTX + PSS/DOXO-coated GNRs@HSA/CS NPs under NIR irradiation (808 nm, 4 min at several power densities) was mainly dominated by an apoptotic pathway (cell apoptotic mortalities ca. 80% in all cases, Fig. 8), as previously observed, for example, for NIR-irradiated gold nanoplates and DOXO-loaded Au nanoshells [78, 79]. In the former case, clear evidences that the molecular apoptotic cascade was mediated by nuclear encoded proteins Bak and Bax through activation of the BH3-only protein BID were provided [79].

In the case of PSS-coated GNRs@HSA/CS hybrid NPs, Annexin V fluorescence increases up to 25% in the absence of light irradiation despite the nanoplatform was fully biocompatible (see above). This behavior might be related to the observed size increment of the latter type of particles under physiological solution conditions, which would induce the beginning of an apoptotic cascade after their internalization [80, 81]. Upon NIR irradiation, apoptotic levels for this type of hybrid NP progressively increase, as also observed for PSS/DOXO-coated GNRs@HSA/CS and DTX + PSS/DOXO-coated GNRs@HSA/CS hybrid NPs. Particularly, necrosis levels are noticeable greater at a fluency of 2 W/cm^2^ (from less than 10% to ca. 40%) as a consequence of extensive membrane blebbing and endosome expansion, as reported elsewhere [36]. Hence, it seems that the type of programmed cell death mechanism depends on several interconnected factors such as the irradiation conditions, type of particle and concentration regime, cellular internalization pathway, amongst others [82].

## Conclusions

In this work, protein-based hybrid nanoparticles encapsulating both DTX and PSS/DOXO-coated GNRs were designed for their potential use as suitable agents for combined PPTT and dual-chemotherapy in the search of potential more efficient breast cancer treatments. The prepared nanohybrids (denoted as DTX + PSS/DOXO-coated GNRs@HSA/CS NPs) aimed to exploit the encapsulation capacity and biocompatibility of HSA and the photothermal properties of GNRs in order to achieve a controlled and modulable release of DOXO and DTX by means of a NIR-light triggering mechanism, which should help in fitting drugs dosage and DOXO/DTX ratio delivered, as demonstrated. In this regard, it was confirmed that DTX was mainly released by simple diffusion out of the protein carrier whilst DOXO release was largely promoted by NIR light irradiation. In addition, the hybrid nanoplatform exhibited good colloidal stability in physiologically relevant media and suitable cell internalization extents thanks to the outer chitosan shell of the hybrid NPs. The bare HSA NPs also display outstanding biocompatibility (> 85% without incorporated drugs in the absence of light irradiation). DTX + PSS/DOXO-coated GNRs@HSA/CS NPs produced larger cytotoxicities in breast MDA-MB-231 cancer cells than the combined dual free administration of DTX + DOXO, reaching toxicities of ca. 90% after 48 h thanks to their sustained drug release from the nanocarrier. Moreover, the attained cytotoxicities are reached using much lower drug concentrations than those required for the administered free dugs. In spite of DOXO + DTX combination therapy displays an antagonistic effect before 24 h of incubation, after 48 h the therapeutic effect becomes largely synergistic and much larger than the dual free drug therapy. This synergistic action can be additionally promoted at 24 h under NIR light laser exposure, with cell toxicities of ca. 85% (2 W/cm^2^ for 4 min). Finally, breast resistant, metastasic cancer cell death is promoted by a predominant apoptotic cell death pathway avoiding complications such as inflammation reactions associated to other mechanisms like cell necrosis.

## Supplementary information

**Additional file 1.** Description of gold nanorod synthesis; procedure for dynamic light scattering and ζ-potential measurements; absorbance spectra of the different nanoplatform components; DTX loading and encapsulation data inside the hybrid nanoplatform; size and ζ-potentials of HSA and HSA/CS NPs encapsulating DTX; absorbance spectra of PSS-coated, PSS/DOXO-coated GNRs and DTX + PSS/DOXO-coated GNRs encapsulated inside HSA/CS NPs; additional fluorescence microscopy images of PSS/DOXO-coated GNRs@HSA/CS NPs; chemotherapeutic effect of DOXO administered as free drug, and of PSS-coated GNRs@HSA/CS and PSS/DOXO-coated GNRs@HSA/CS NPs; and combination indices. Additional details on GNR synthesis, DLS and ζ-potential data, GNR characterization and cargo encapsulation, and additional figures.

## References

[1] Moulder S, Hortobagyi GN (2008). Advances in the treatment of breast cancer. Clin Pharmacol Ther.

[2] https://www.cancer.gov/types/breast/patient/breast-treatment-pdq#section/all. Accessed April 2019.

[3] Tanaka T, Decuzzi P, Cristofanilli M, Sakamoto JH, Tasciotti E, Robertson FM, Ferrari M (2009). Nanotechnology for breast cancer therapy. Biomed Microdevices.

[4] Persidis A (1999). Cancer multidrug resistance. Nat Biotechnol.

[5] Al-Hajj M, Wicha MS, Benito-Hernandez A, Morrison SJ, Clarke MF (2003). Prospective identification of tumorigenic breast cancer cells. Proc Natl Acad Sci USA.

[6] Ramaswamy SN (2007). Rational design of cancer-drug combinations. N Engl J Med.

[7] Hasenstein JR, Shin HC, Kasmerchak K, Buehler D, Kwon GS, Kozak KR (2012). Antitumor activity of triolimus: a novel multidrug-loaded micelle containing paclitaxel, rapamycin, and 17-AAG. Mol Cancer Ther.

[8] Shin HC, Alani AW, Cho H, Bae Y, Kolesar JM, Kwon GS (2011). A 3-in-1 polymeric micelle nanocontainer for poorly water-soluble drugs. Mol Pharm.

[9] Bae Y, Alani AW, Rockich NC, Lai TS, Kwon GS (2010). Mixed pH-sensitive polymeric micelles for combination drug delivery. Pharm Res.

[10] Mishra GP, Nguyen D, Alani AWG (2013). Inhibitory effect of paclitaxel and rapamycin individual and dual drug-loaded polymeric micelles in the angiogenic cascade. Mol Pharm.

[11] Navarro G, Pan J, Torchilin VP (2015). Micelle-like nanoparticles as carriers for DNA and siRNA. Mol Pharm.

[12] Xie J, Lee S, Chen X (2010). Nanoparticle-based theranostic agents. Adv Drug Deliv Rev.

[13] Danhier F, Ansorena E, Silva JM, Coco R, Le Breton A, Préat V (2012). PLGA-based nanoparticles: an overview of biomedical applications. J Control Release.

[14] Villar-Alvarez E, Figueroa-Ochoa E, Barbosa S, Soltero JFA, Taboada P, Mosquera V (2015). Reverse poly(butylene oxide)–poly(ethylene oxide)–poly(butylene oxide) block copolymers with lengthy hydrophilic blocks as efficient single and dual drug-loaded nanocarriers with synergistic toxic effects on cancer cells. RSC Adv.

[15] Weissig V, Pettinger TK, Murdock N (2014). Nanopharmaceuticals (part 1): products on the market. Int J. Nanomed.

[16] Zhang D, Kong YY, Sun JH, Huo SJ, Zhou M, Gui YL, Mu X, Chen H, Yu SQ, Xu Q (2017). Co-delivery nanoparticles with characteristics of intracellular precision release drugs for overcoming multidrug resistance. Int J Nanomed.

[17] Wu C, Gong MQ, Liu BY, Zhuo RX, Cheng SX (2017). Co-delivery of multiple drug resistance inhibitors by polymer/inorganic hybrid nanoparticles to effectively reverse cancer drug resistance. Colloids Surf B Biointerfaces.

[18] Yin F, Yang C, Wang Q, Zeng S, Hu R, Lin G, Tian J, Hu S, Lan RF, Yoon H, Lu F, Wang K, Yong K-T (2015). A light-driven therapy of pancreatic adenocarcinoma using gold nanorods-based nanocarriers for co-delivery of doxorubicin and siRNA. Theranostics.

[19] Chen AM, Zhang M, Wei D, Stueber D, Taratula O, Minko T, He H (2009). Co-delivery of doxorubicin and Bcl-2 siRNA by mesoporous silica nanoparticles enhances the efficacy of chemotherapy in multidrug-resistant cancer cells. Small.

[20] Jain RK (1998). The next frontier of molecular medicine: delivery of therapeutics. Nat Med.

[21] Sengupta S, Eavarone D, Capila I, Zhao G, Watson N, Kiziltepe T, Sasisekharan R (2005). Temporal targeting of tumour cells and neovasculature with a nanoscale delivery system. Nature.

[22] Gonzalez-Angulo AM, Morales-Vasquez F, Hortobagyi GN (2007). Overview of resistance to systemic therapy in patients with breast cancer. Adv Exp Med Biol.

[23] Ariffin AB, Forde PF, Jahangeer S, Soden DM, Hinchion J (2014). Releasing pressure in tumors: what do we know so far and where do we go from here? A review. Cancer Res.

[24] Topete A, Melgar D, Alatorre-Meda M, Iglesias P, Argibay B, Vidawati S, Barbosa S, Costoya JA, Taboada P, Mosquera V (2014). NIR-light active hybrid nanoparticles for combined imaging and bimodal therapy of cancerous cells. J Mater Chem B.

[25] Sapsford KE, Russ Algar W, Berti L, Boeneman-Gemmill K, Casey BJ, Oh E, Stewart MH, Medintz IL (2013). Functionalizing nanoparticles with biological molecules: developing chemistries that facilitate nanotechnology. Chem Rev.

[26] Hu M, Chen J, Li ZY, Au L, Hartland GV, Li X, Marquez M, Xia Y (2006). Gold nanostructures: engineering their plasmonic properties for biomedical applications. Chem Soc Rev.

[27] Ali MRK, Rahman MA, Wu Y, Han T, Peng X, Mackey MA, Wang D, Shin HJ, Chen ZG, Xiao H, Wu R, Tang Y, Shin DM, El-Sayed MA (2017). Efficacy, long-term toxicity, and mechanistic studies of gold nanorods photothermal therapy of cancer in xenograft mice. Proc Natl Acad Sci USA.

[28] Jain PK, Huang XH, El-Sayed IH, El-Sayed MA (2008). Noble metals on the nanoscale: optical and photothermal properties and some applications in imaging. Acc Chem Res.

[29] Yamashita S, Fukushima H, Akiyama Y, Niidome Y, Mori T, Katayama Y, Niidome T (2011). Controlled-release system of single-stranded DNA triggered by the photo-thermal effect of gold nanorods and its in vivo applica-tion. Bioorg Med Chem.

[30] Radaideh SM, Sledge GW (2008). Taxane vs. taxane: is the duel at an end? A commentary on a phase-III trial of doxorubicin and docetaxel versus doxorubicin and paclitaxel in metastatic breast cancer: results of the ERASME 3 study. Breast Cancer Res Treat.

[31] Kratz F (2014). A clinical update of using albumin as a drug vehicle. A commentary. J Control Release.

[32] Kunjachan S, Jose S, Lammers T (2010). Understanding the mechanism of ionic gelation for synthesis of chitosan nanoparticles using qualitative techniques. Asian J Pharm.

[33] Encinas-Basurto D, Ibarra J, Juarez J, Pardo A, Barbosa S, Taboada P, Valdez MA (2018). Hybrid folic acid-conjugated gold nanorods-loaded human serum albumin nanoparticles for simultaneous photothermal and chemotherapeutic therapy. Mater Sci Eng C.

[34] Robles E, Villar E, Alatorre-Meda M, Burboa MG, Valdez MA, Taboada P, Mosquera V (2013). Effects of the hydrophobization on chitosan-insulin nanoparticles obtained by an alkylation reaction on chitosan. J Appl Polym Sci.

[35] Kantner K, Rejman D, Kraft KVL, Soliman MG, Zyuzin MV, Escudero A, del Pino P, Parak WJ (2018). Laterally and temporally controlled intracellular staining by light-triggered release of encapsulated fluorescent markers. Chem Eur J.

[36] Chou TC, Martin N (2007). CompuSyn software for drug combinations and for general dose-effect analysis, and user’s guide.

[37] Chou TC, Talalay P (1984). Quantitative analysis of dose-effect relationships: the combined effects of multiple drugs or enzyme inhibitors. Adv Enzyme Regul.

[38] Robles E, Juárez J, Burboa MG, Gutiérrez LE, Taboada P, Mosquera V, Valdez MA (2014). Properties of insulin-chitosan complexes obtained by an alkylation reaction on chitosan. J Appl Polym Sci.

[39] Kim K, Kwon S, Park JH, Chung H, Jeong SY, Kwon IC, Kim IS (2005). Physicochemical characterizations of self-assembled nanoparticles of glicol chitosan-deoxycholic acid conjugates. Biomacromolecules.

[40] Bronze-Uhle E, Costa BC, Ximenes VF, Lisboa-Filho PN (2016). Synthetic nanoparticles of bovine serum albumin with entrapped salicylic acid. Nanotechnol Sci Appl.

[41] Satya Prakash S (2010). Human serum albumin nanoparticles as an efficient noscapine drug delivery system for potential use in breast cancer: preparation and in vitro analysis. Int J Nanomed.

[42] Morris GA, Castile J, Smith A, Adams GG, Harding SE (2011). The effect of prolonged storage at different temperatures on the particle size distribution of tripolyphosphate (TPP)–chitosan nanoparticles. Carbohydr Polym.

[43] Chen R, Zheng X, Qian H, Wang X, Wang J, Jiang X (2013). Combined near-IR photothermal therapy and chemotherapy using gold-nanorod/chitosan hybrid nanospheres to enhance the antitumor effect. Biomater Sci.

[44] Meng Z, Wei F, Wang R, Xia M, Chen Z, Wang H, Zhu M (2016). NIR-laser switched in vivo smart nanocapsules for synergic photothermal and chemotherapy of tumors. Adv Mater.

[45] Pustovalov VK (2016). Light-to-heat conversion and heating of single nanoparticles, their assemblies, and the surrounding medium under laser pulses. RSC Adv.

[46] Barbosa S, Topete A, Alatorre-Meda M, Villar-Alvarez EM, Pardo A, Alvarez-Lorenzo C, Concheiro A, Taboada P, Mosquera V (2014). Targeted combinatorial therapy using gold nanostars as theranostic platforms. J Phys Chem C.

[47] Liu Y, Zhi X, Yang M, Zhang J, Lin L, Zhao X, Hou W, Zhang C, Zhang Q, Pan F, Alfranca G, Yag Y, de la Fuente JM, Ni J, Cui D (2017). Tumor-triggered drug release from calcium carbonate-encapsulated gold nanostars for near-infrared photodynamic/photothermal combination antitumos therapy. Theranostics.

[48] Song J, Yang X, Yang Z, Lin L, Liu Y, Zhou Z, Shen Z, Yu G, Dai Y, Jacobson O, Munasinghe J, Yung B, Teng G-J, Chen X (2017). Rational design of branched nanoporous gold nanoshells with enhanced physico-optical properties for optical imaging and cancer therapy. ACS Nano.

[49] Huang S, Duan S, Wang J, Bao S, Qiu X, Li C, Liu Y, Yan L, Zhang Z, Hu Y (2016). Folic-acid-mediated functionalized gold nanocages for targeted delivery of anti-miR-181b in combination of gene therapy and photothermal therapy against hepatocellular carcinoma. Adv Funct Mater.

[50] Bao C, Conde J, Pan F, Zhang C, Tian F, Liang S, de la Fuente JM, Cui D (2016). Gold nanoprisms as a hybrid in vivo cancer theranostic platform for in situ photoacoustic imaging, angiography and localized hyperthermia. Nano Res.

[51] Topete A, Alatorre-Meda M, Villar-Alvarez EM, Carregal-Romero S, Barbosa S, Parak WJ, Taboada P, Mosquera V (2014). Polymeric-gold nanohybrids for combined imaging and cancer therapy. Adv Health Mater.

[52] Zhan C, Wang W, McAlvin JB, Guo S, Timko BP, Santamaria C, Kohane DS (2016). Phototriggered local anesthesia. Nano Lett.

[53] Li M, Sun X, Zhang N, Wang W, Yang Y, Jia H, Liu W (2018). NIR-activated polydopamine-coated carrier-free “nanobomb” for in situ-on-demand drug release. Adv Sci.

[54] Espinosa A, Di Corato R, Kolsnjaj-Tabi J, Flaud P, Pellegrino T, Wilhelm C (2016). Duality of iron oxide nanoparticles in cancer therapy: amplification of heating efficiency by magnetic hyperthermia and photothermal bimodal treatment. ACS Nano.

[55] Chen H, Song M, Tang J, Hu G, Xu S, Guo Z, Li N, Cui J, Zhang X, Chen X, Wang L (2016). Ultrahigh ^19^F loaded Cu_1.75_S nanoprobes for simultaneous ^19^F magnetic resonance imaging and photothermal therapy. ACS Nano.

[56] Zhang B, Wang H, Shen S, She X, Shi W, Chen J, Zhang Q, Hu Y, Pang Z, Jiang X (2016). Fibrin-targeted peptide CREKA-conjugated multi-walled carbon nanotubes for self-amplified photothermal therapy of tumor. Biomaterials.

[57] Ma H, Jiang C, Zhai D, Luo Y, Chen Y, Lu F, Yi Z, Deng Y, Wang J, Chang J, Wu C (2016). A bifunctional biomaterial with photothermal effect for tumor therapy and bone regeneration. Adv Funct Mater.

[58] Yu L, Dong A, Guo R, Yang M, Deng L, Zhang J (2018). DOX/ICG coencapsulated liposome-coated thermosensitive nanogels for NIR-triggered simultaneous drug release and photothermal effect. ACS Biomater Sci Eng.

[59] Li X, Wang X, Zhao C, Shao L, Lu J, Tong Y, Chen L, Cui X, Sun H, Liu J, Li M, Deng X, Wu Y (2019). From one to all: self-assembled theranostic nanoparticles for tumor-targeted imaging and programmed photoactive therapy. J Nanobiotechnol.

[60] Deng L, Cai X, Sheng D, Yang Y, Strohm EM, Wang Z, Ran H, Wang D, Zeng Y, Li P, Shang T, Ling Y, Wang F, Sun Y (2017). A laser-activated biocompatible theranostic nanoagent for targeted multimodal imaging and photothermal therapy. Theranostics.

[61] Nourhashemi M, Mahmoudzadeh M, Wallois F (2016). Thermal impact of near-infrared laser in advanced noninvasive optical brain imaging. Neurophotonics.

[62] Al-Qadi S, Alatorre-Meda M, Martin-Pastor M, Taboada P, Remuñán-López C (2016). The role of hyaluronic acid inclusion on the energetics of encapsulation and release of a protein molecule from chitosan-based nanoparticles. Colloids Surf B Biointerfaces.

[63] Cambón A, Rey-Rico A, Mistry D, Brea J, Loza MI, Attwood D, Barbosa S, Alvarez-Lorenzo C, Concheiro A, Taboada P, Mosquera V (2013). Cytocompatibility and P-glycoprotein inhibition of block copolymers: structure–activity relationship. Int J Pharm.

[64] Cabeza L, Ortiz R, Arias JL, Prados J, Ruiz-Martínez MA, Entrena JM, Luque R, Melguizo C (2015). Enhanced antitumor activity of doxorubicin in breast cancer through the use of poly(butylcyanoacrylate) nanoparticles. Int J Nanomed.

[65] Pikabea A, Villar-Alvarez EM, Forcada J, Taboada P (2018). pH-controlled doxorubicin delivery from PDEAEMA-based nanogels. J Mol Liq.

[66] Duan W, Liu Y (2018). Targeted and synergistic therapy for hepatocellular carcinoma:monosaccharide modified lipid nanoparticle for the co-delivery of doxorubicin and sorafenib. Drug Des Dev Ther.

[67] Li C, Lai C, Qiu Q, Luo X, Hu L, Zheng H, Lu Y, Liu M, Zhang H, Liu X, Deng Y, Song Y (2019). Dual-ligand modification of PEGylated liposomes used for targeted doxorubicin delivery to enhance anticancer efficacy. AAPS PharmSciTech.

[68] Wang T, Jiang Y, Chu H, Liu X, Dai Y, Wang D (2019). Doxorubicin and lovastatin co-delivery liposomes for synergistic therapy of liver cancer. J Drug Deliv Sci Technol.

[69] Kuo Y-C, Chang Y-H, Rajesh R (2019). Targeted delivery of etoposide, carmustine and doxorubicin to human glioblastoma cells using methoxy poly(ethylene glycol)–poly(ε-caprolactone) nanoparticles conjugated with wheat germ agglutinin and folic acid. Mater Sci Eng C.

[70] Qu Q, Wang Y, Zhang L, Zhang X, Zhou S (2016). A nanoplatform with precise control over release of cargo for enhanced cancer therapy. Small.

[71] Yen H-C, Cabral H, Mi P, Toh K, Matsumoto Y, Liu X, Koori H, Kim A, Miyazaki K, Miura Y, Nishiyama N, Kataoka K (2014). Light-induced cytosolic activation of reduction-sensitive camptothecin-loaded polymeric micelles for spatiotemporally controlled in vivo chemotherapy. ACS Nano.

[72] Huschka R, Zuloaga J, Knight MW, Brown LV, Nordlander P, Halas NJ (2011). Light-induced release of DNA from gold nanoparticles: nanoshells and nanorods. J Am Chem Soc.

[73] Jiang Q, Song C, Nangreave J, Liu X, Lin L, Qiu D, Wang ZG, Zou G, Liang X, Yan H, Ding B (2012). DNA origami as a carrier for circumvention of drug resistance. J Am Chem Soc.

[74] Lakkadwala S, dos Santos Rodrigues B, Sun C, Singh J (2019). Dual functionalized liposomes for efficient co-delivery of anticancer chemotherapeutics for the treatment of glioblastome. J Control Release.

[75] Strozyk MS, Carregal-Romero S, Henriksen-Lacey M, Brust M, Liz-Marzán LM (2017). Biocompatible, multiresponsive nanogel composites for codelivery of antioangiogenic and chemotherapeutic agents. Chem Mater.

[76] Zhao M, Bozzato E, Joudiou N, Ghiassinejad S, Danhier F, Gallez B, Préat V (2019). Codelivery of paclitaxel and temozolomide through a photopolymerizable hydrogel prevents glioblastome recurrence after surgical resection. J Control Release.

[77] Kenmotsu H, Tanigawara Y (2015). Pharmacokinetics, dynamics and toxicity of docetaxel: why the Japanese dose differs from the Western dose. Cancer Sci.

[78] Rowe-Horwege RW, Webster JG (2006). Systemic Hyperthermia. Encyclopedia of medical devices and instrumentation.

[79] Pérez-Hernández M, del Pino P, Mitchell SG, Moros M, Stepien G, Pelaz B, Parak WJ, Gálvez EM, Pardo J, de la Fuente JM (2015). Dissecting the molecular mechanism of apoptosis during photothermal therapy using gold nanoprisms. ACS Nano.

[80] Topete A, Alatorre-Meda M, Iglesias P, Villar-Alvarez EM, Barbosa S, Costoya JA, Taboada P, Mosquera V (2014). Fluorescent drug-loaded polymeric-based branched gold nanoshells for localized multimodal therapy and imaging of tumoral cells. ACS Nano.

[81] Caster JM, Yu SK, Patel AN, Newman NJ, Lee ZJ, Warner SB, Wagner KT, Roche KC, Tian X, Min Y, Wang AA (2017). Effect of particle size on the biodistribution, toxicity, and efficacy of drug-loaded polymeric nanoparticles in chemoradiotherapy. Nanomed Nanotechnol Biol Med.

[82] Fernández-Cabada T, Lopez de Pablo CS, Serrano AM, del Pozo-Guerrero FP, Serrano-Olmedo JJ, Gómez MR (2012). Induction of cell death in a glioblastome line by hyperthermia therapy based on gold nanorods. Int J Nanomed.

