# Supplementary material for: Combination of light-driven co-delivery of chemodrugs and plasmonic-induced heat for cancer therapeutics using hybrid protein nanocapsules
Source: J Nanobiotechnology. 2019 Oct 15;17:106. doi: 10.1186/s12951-019-0538-3 (PMC6794818; doi:10.1186/s12951-019-0538-3)
Supplement: Supplementary file 1 — Additional file 1. Description of gold nanorod synthesis; procedure for dynamic light scattering and ζ-potential measurements; absorbance spectra of the different nanoplatform components; DTX loading and encapsulation data inside the hybrid nanoplatform; size and ζ-potentials of HSA and HSA/CS NPs encapsulating DTX; absorbance spectra of PSS-coated, PSS/DOXO-coated GNRs and DTX + PSS/DOXO-coated GNRs encapsulated inside HSA/CS NPs; additional fluorescence microscopy images of PSS/DOXO-coated GNRs@HSA/CS NPs; chemotherapeutic effect of DOXO administered as free drug, and of PSS-coated GNRs@HSA/CS and PSS/DOXO-coated GNRs@HSA/CS NPs; and combination indices. Additional details on GNR synthesis, DLS and ζ-potential data, GNR characterization and cargo encapsulation, and additional figures. [file 12951_2019_538_MOESM1_ESM.docx]

**SUPPORTING INFORMATION FOR**

**Combination of light-driven co-delivery of chemodrugs and plasmonic-induced heat for cancer therapeutics using hybrid protein nanocapsules**

E. Villar-Alvarez^1*^, A. Cambón^1^, A. Pardo^1^, L. Arellano^1^, A. V. Marcos^1^, B. Pelaz^1,2^, P. del Pino^1,2^, A. Bouzas-Mosquera^3^, V.X. Mosquera^3^, A. Almodlej^4^, G. Prieto^5^, S. Barbosa^1,6^, P. Taboada ^1,6*^

^1^Grupo de Física de Coloides y Polímeros, Departamento de Física de la Materia Condensada; Universidad de Santiago de Compostela, 15782-Santiago de Compostela, Spain.

^2^Centro Singular de Investigación en Química Biológica y Materiales Moleculares (CiQUS), Universidad de Santiago de Compostela, 15782-Santiago de Compostela, Spain.

^3^Departamento de Cirugía Cardíaca, Complexo Hospitalario Universitario A Coruña, Instituto de Investigación Biomédica de A Coruña (INIBIC), A Coruña, Spain.

^4^Department of Physics and Astronomy, College of Science, King Saud University, Riyadh 11451, Saudi Arabia

^5^Grupo de Biofísica e Interfases, Departamento de Física Aplicada; Universidad de Santiago de Compostela, 15782-Santiago de Compostela, Spain.

^6^Instituto de Investigaciones Sanitarias (IDIS) y Agrupación Estratégica de Materiales, Universidad de Santiago de Compostela, 15782-Santiago de Compostela, Spain.

^*^Author to whom correspondence should be addressed: [eva.mailbox1@ugmail.com](mailto:eva.mailbox1@ugmail.com); pablo.taboada@usc.

**S1. Synthesis of gold nanorods (GNRs)**

Firstly, CTAB-capped Au seeds were obtained. To do that, 7.5 mL of a 0.2 M CTAB solution was gently mixed with 0.25 mL of 0.01 M HAuCl_4_ in a water bath at 27 ºC. As an indication, for both seeds and GNRs preparation, CTAB was left at a constant temperature of 27 ºC for one day under constant stirring of 200 rpm to get its full solubilisation and avoid foaming prior to use. Next, a 0.01 M NaBH_4_ solution was prepared in ice-cold water. This solution was left to rest for 2-3 min to ensure a good dispersion of the reductant. Afterwards, while the Au-CTAB solution was stirred at 200 rpm, 0.6 mL of ice-cold 0.01 M NaBH_4_ were added in one pull to the former, after which the mixed solution turned brownish yellow. This was mixed gently by hand for 2 min and then left undisturbed in a water bath at 27º C for 1 h to allow the excess sodium borohydride to be decomposed.

For the growth of GNRs, 425 µL of a 0.01 M HAuCl_4_ solution were added to 10 mL of 0.2 M CTAB in a water bath at 27 ºC, after which the solution turned yellow bright while stirred at 500 rpm. Then, 0.01 M of an AgNO_3_ solution was prepared in the dark. Different volumes of the silver solution (ranging from 63 µL to 143 µL) were added to the Au growth solution followed by gentle mixing by hand. Then, 68 µL of a 0.1 M ascorbic acid (AA) solution were added followed by gentle stirring at 500 rpm until the solution turned colourless, and left it in this way for 4 min in a water bath at 27 ºC. Finally, 110 µL of the Au seed solution were gently added to the Au growth solution while stirring for 2 min and, then, stopped. The resulting solution was left undisturbed in a water bath overnight at 27 ºC becoming reddish-pink. The formed GNRs were centrifuged at least twice at 27 ºC for 20 min and redispersed in 10 mL of deionized water. The UV- visible absorption spectra of the obtained GNRs were measured using a Cary Bio 100 UV-vis spectrophotometer (Agilent Technologies, USA). The sizes and ARs of the GNRs were measured using a JEOL JEM 1011 (Japan) transmission electron microscope operating at an accelerating voltage of 120 kV.

**S2. Dynamic light scattering and ζ-potential measurements**.

Dynamic light scattering (DLS) was performed by means of an ALV-5000F (ALV-GmbH, Germany) instrument with vertically polarized incident light (λ = 488 nm) supplied by a diode-pumped Nd:YAG solid-state laser (Coherent Inc., CA, USA) operated at 2 W, and combined with an ALV SP-86 digital correlator with a sampling time of 25 ns to 100 ms. The intensity scale was calibrated against scattering from toluene. Measurements were made at a scattering angle *θ* = 90° to the incident beam. Solutions were allow to equilibrate for 5 min before measurement. Experiment duration was in the range 2-5 min, and each experiment was repeated at least three times. The correlation functions from DLS runs were analyzed by the CONTIN method [S1] to obtain the intensity distributions of decay rates (*Γ*). From the decay rate distributions, the apparent diffusion coefficients (*D_app_* = Γ/*q*2, *q* = (4π*n*s/λ)sin(θ/2)) were derived, being *n*s the refractive index of the solvent. Values of the apparent hydrodynamic radius (*R_H_*, radius of the hydrodynamically equivalent hard sphere corresponding to *D_app_*) were calculated from the Stokes-Einstein equation:

 (S1)

where *k_B_* is the Boltzmann constant*, T* the temperature, and *η* the solvent viscosity. Solutions were filtered with 5 μm syringe filters to avoid the presence of very large aggregates/complexes out of the range of the DLS instrument.

ζ-potentials of protein-based hybrid NPs were measured using a Nano ZS-90 instrument (Nanoseries, Malvern Instruments, UK). The equipment measured the electrophoretic mobility of the particles and converted it to the ζ-potential using the classical Smoluchowski equation with the Smoluchowski approximation (aqueous media and moderate electrolyte concentration):

$\alpha=\varepsilon\frac{\zeta}{\eta}$(S2)

where α, ε, ζ, and η denote the electrophoretic mobility, permittivity of the media, ζ-potential of the particles, and viscosity of the media, respectively. Each sample was loaded into a folded capillary, clear, disposable cell. Measurements were initiated after attaining thermal equilibrium at 25 ºC. The number of runs for each measurement was automatically determined by the software but always larger than 20. The measurements were performed by triplicate. Results were reported as the mean ± standard deviation (SD).

**S3. PSS/DOXO-coated GNRs**

Absorbance spectra of GNRs (33 × 9 nm) loaded with DOXO are shown in Figure S1. GNRs display two localized surface plasmon resonance (LPSR) bands: the transversal one at ca. 510 nm, and the longitudinal one at ca. 780 nm. A characteristic peak is observed at 490 nm, denoting the successful attachment of DOXO on the GNR surfaces. This peak is enhanced by means of the coupling of the DOXO absorbance band with the transversal mode of the metallic particles.

It is well-known that the surface plasmon resonance bands of metallic NPs are sensible to changes in their local dielectric surrounding environment [S2]. Thus, the adsorption of different species will cause a shift in the LSPR bands. In fact, the PSS coating produces a blue-shift since the refractive index (RI) of this polymer (1.38) is lower than that of the original CTAB layer resulting for the synthetic procedure (1.435) [S3]. Conversely, RI of DOXO is larger (1.71) than that of PSS, so a red shift would be expected, as observed. In addition, the light absorbance abruptly drops after the adsorption of the PSS layer. This damping is a consequence of the decrease in the amplitude of electron oscillations along the longitudinal axis as a consequence of the transfer of hot electrons to the wrapped polyelectrolyte coating [S4], that is, part of the incoming light energy is absorbed by the polymeric layer, slowing down the longitudinal electron oscillations and decreasing the LSPR [S5].

**
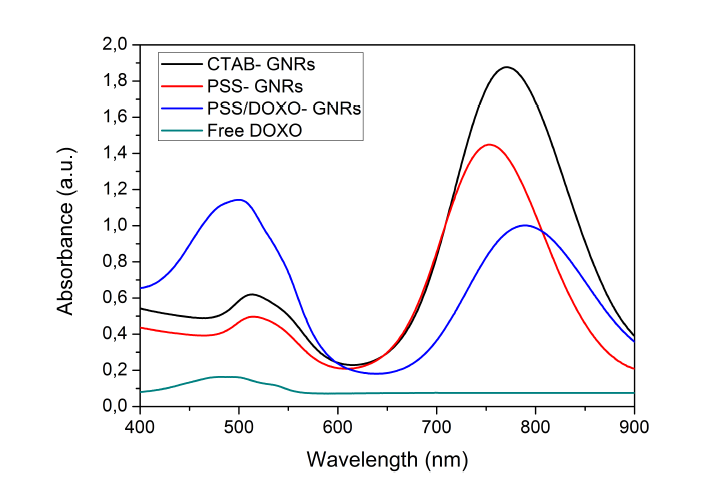
**

**Figure S1**: Absorbance spectra of bare (**^______^**), PSS-coated (**^______^**), PSS/DOXO-coated GNRs (**^______^**) and free DOXO (**^______^**).

**S4. Loading of DTX inside hybrid HSA/CS NPs**

To provide a dual combined chemotherapy using the present hybrid HSA/CS NPs, the anticancer drug DTX was additionally loaded inside the hybrid protein-based NPs. The use of DTX is limited by its poor aqueous solubility, low bioavailability, high toxicity, with its currently marketed form (Taxotere®) for intravenous infusion is formulated utilizing Tween 80 and ethanol, which leads to toxic side effects and hypersensitivity after administration [S6,S7]. Thus, the incorporation of this drug inside a nanovehicle can preclude some of these problems as well as to provide an additional complementary chemotherapeutic outcome to that of DOXO when combined in a single treatment.

Different initial amounts of DTX were loaded inside bare, PSS-coated and PSS/DOXO-coated GNRs loaded inside HSA (DTX+PSS-coated GNRs@HSA and DTX+PSS/DOXO-coated GNRs@HSA NPs, respectively) and HSA/CS NPs (DTX+PSS-coated GNRs@HSA/CS and DTX+PSS/DOXO-coated GNRs@HSA/CS NPs, respectively). No significant changes in particle sizes and surface electrical charge were found upon incorporation of DTX (50 to 200 μg/mL) except for bare HSA NPs, for which a certain size reduction was observed (see Figure S3 below). Also, the extent of DTX encapsulation increased as the initial fed amount did. Figure S2a shows that at a low initial fed drug concentration (< 100 μg/mL) the mass of loaded DTX inside bare HSA and PSS/DOXO-GNRs@HSA NPs is ca. 15 µg and, then, progressively increases, especially for the latter type of NPswith values of 114 and 198 μg, 400 and 700 times the aqueous solubility of free DTX, respectively. As more DTX is added, hydrophobic interactions between drug molecules become predominant, so DTX incorporation inside the core of the protein particles is largely favored. Moreover, the larger core volume of PSS/DOXO-GNRs@HSA NPs as well as the possible existence of additional interactions between the DOXO-functionalised GNRs and DTX might additionally favor DTX loading [S8]. In this manner, loading capacities (LCs) of ca. 3 and 5% and encapsulation efficiencies (EEs) of up to ca. 20 and 40% can be obtained for HSA and PSS/DOXO-GNRs@HSA NPs, respectively, as observed in Figure S2b-c.





|  |  |  |
| --- | --- | --- |

**Figure S2**: (a) Amount of DTX inside bare HSA and PSS/DOXO-GNRs@HSA NPs; (b) entrapment efficiency (EE), and (c) drug loading capacity (LC) of DTX as a function of the initial fed drug.

**S.4. Size and ξ-potentials of HSA and HSA/CS NPs encapsulating DTX.**

The diameter of bare HSA linearly increases as the concentrations of added DTX changes from 50 to 500 µg (Figure S5a). Upon incorporation of DTX, HSA NPs ζ-potentials (Figure S5b) remain negative with a slight increase as a result of the exposure of carboxylic acid groups of protein residues to the aqueous medium to create a more hydrophobic particle core to facilitate the incorporation of the drug.


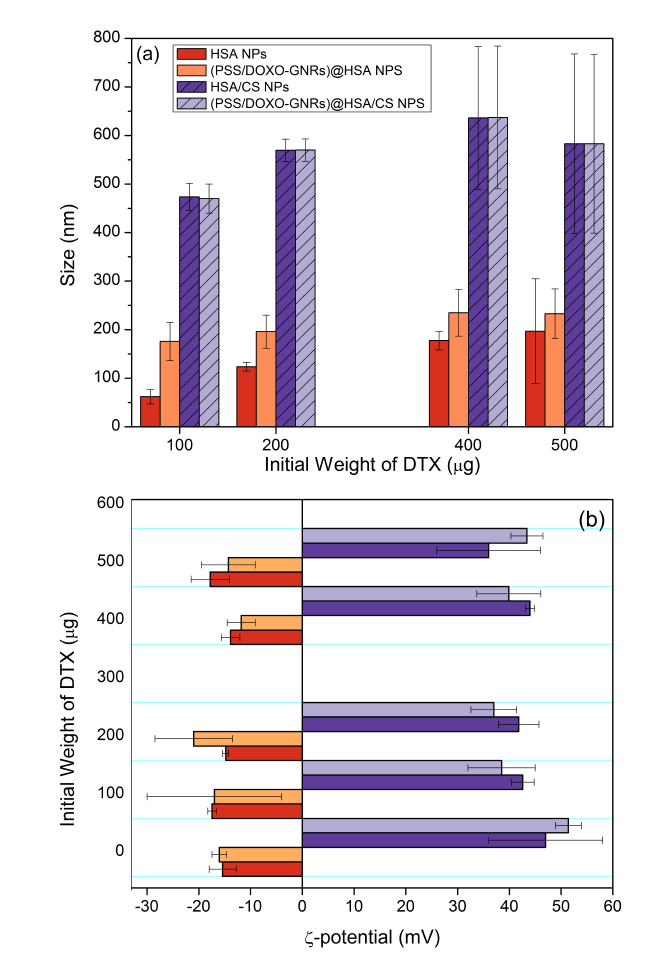


**Figure S3**: (a) Particle sizes and (b) ζ-potentials of bare and PSS/DOXO-coated GNRs-loaded HSA and HSA/CSNPs encapsulating different initial amounts of DTX.

In contrast, hybrid (PSS/DOXO-coated GNRs@HSA NPs particle sizes remains barely constant as the initial amount of DTX is increased (Figure S3a). When chitosan is used to coat the NPs larger sizes (ca. 300 nm) are observed. Surprisingly upon the addition of DTX no size variations between (PSS/DOXO-coated GNRs@HSA/CS and bare HSA/CS NPs are observed probably as a consequence of the formation of a thinner biopolymer shell (that is, as the particle size becomes larger more chitosan is required to cover the NP surface so a thinner shell is formed). Nonetheless, it can be observed that for PSS/DOXO-coated GNRs-@HSA/CS particles, sizes at high initial DTX loading are ca. 550 nm, much larger than those corresponding to DTX-encapsulated bare HSA NPs. On the other hand, ζ-potential data (Figure S3b) are barely constant at ca. 40-50 mV independently of the presence or not of encapsulated PSS/DOXO-coated GNRs within the particle.

**S5. Spectral properties of PSS-coated, PSS/DOXO-coated GNRs and DTX+PSS/DOXO-coated GNRs encapsulated inside HSA/CS NPs.**

Figures S4a-b shows the UV-vis spectra of HSA and HSA/CS NPs loaded with PSS-coated and PSS/DOXO-coated GNRs. Figure S5a-b shows the UV-vis spectra of HSA and HSA/CS NPs loaded with DTX in the presence and absence of PSS/DOXO-coated GNRs in their inner cores. A peak at 230 nm characteristic of DTX can be observed (after several washing steps) confirming the DTX is located within the protein-based hybrid NPs.

**
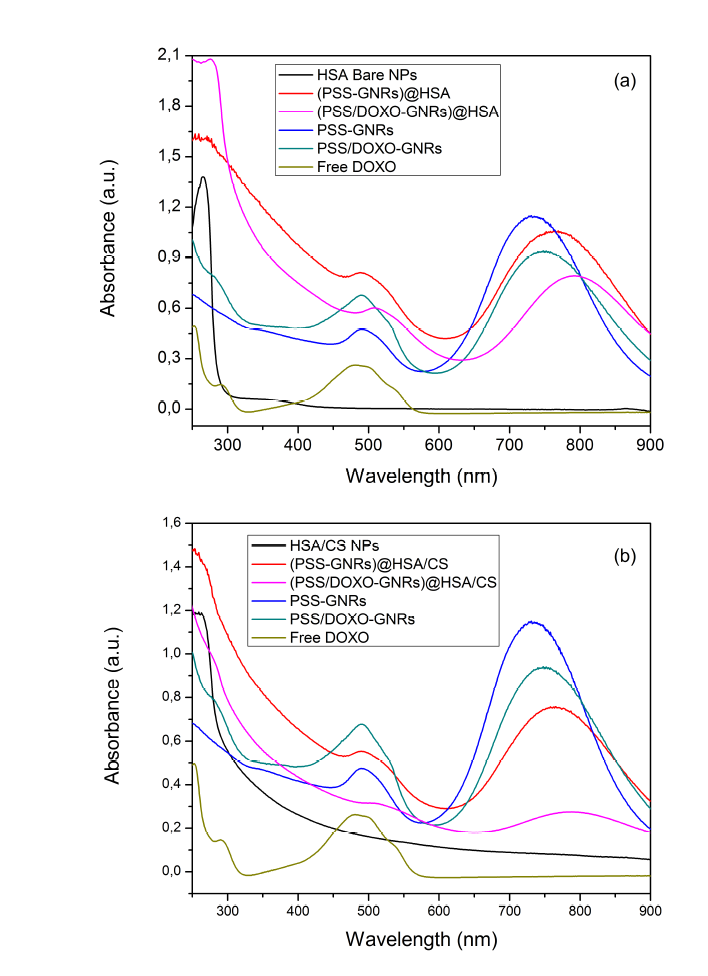
**

**Figure S4**: UV-vis spectra of (a) HSA and (b) HSA/CS NPs without (^__^) and with encapsulated PSS-coated (^__^) or PSS/DOXO-coated GNRs (^__^). Free DOXO (^__^), PSS-coated (^__^) and PSS/DOXO-coated GNRs (^__^) spectra are also shown as references.

**
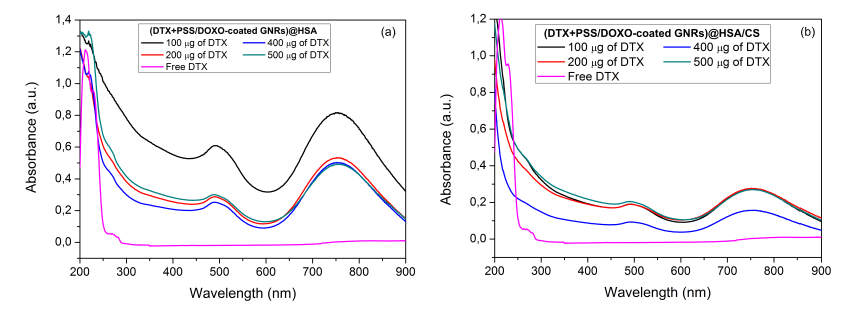
**

**Figure S5**: UV-vis spectra data of (a) HSA and (b) HSA/CS NPs loaded with PSS/DOXO-coated GNRs and several amounts of DTX.

**S6. Fluorescence microscopy images of PSS/DOXO-coated GNRs@HSA/CS NPs.**

**
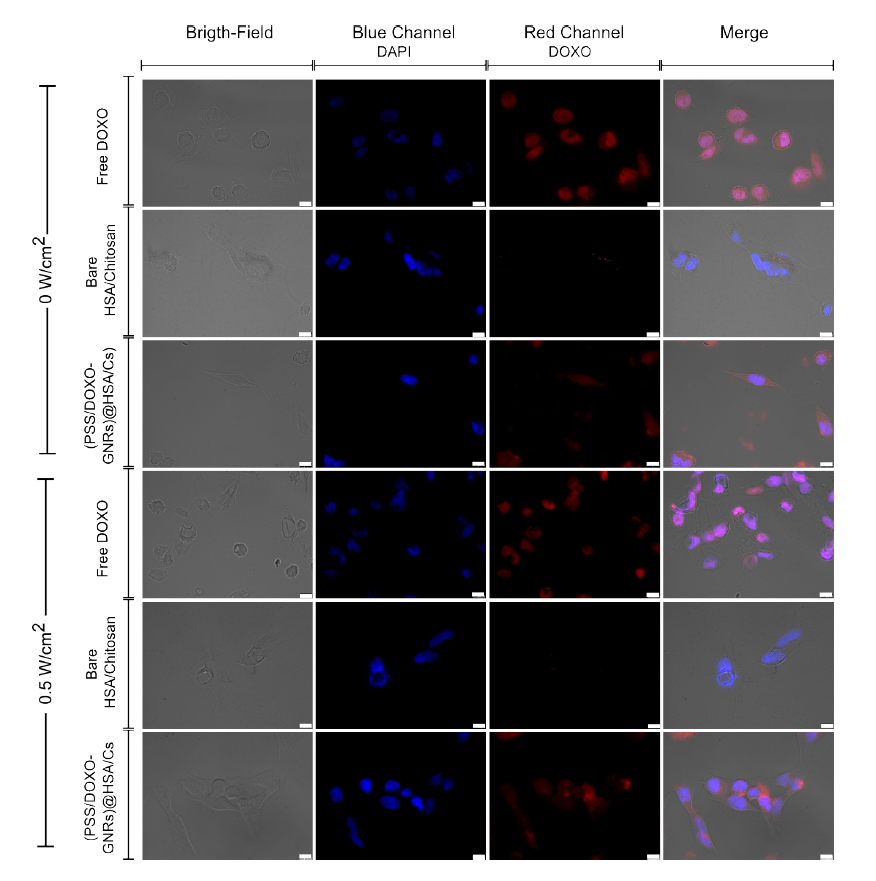
**

**Figure S6**: Fluorescence microscopy images of hybrid NP cellular uptake and subsequent intracellular DOXO release in MDA-MB-231 cells. Free DOXO, bare HSA/CS and PSS/DOXO-coated GNRs@HSA/CS NPs are shown after 8 h of incubation in the presence and absence of NIR light illumination (0.5 W/cm^2^ at 808 nm for 5 min after 6 h of incubation). Red channel was from DOXO fluorescence (λ_exc_ = 488 nm); blue channel from cell nuclei stained with DAPI (λ_exc_ =355 nm); bright field channel and merged images are also shown. Scale bar is 10 µm


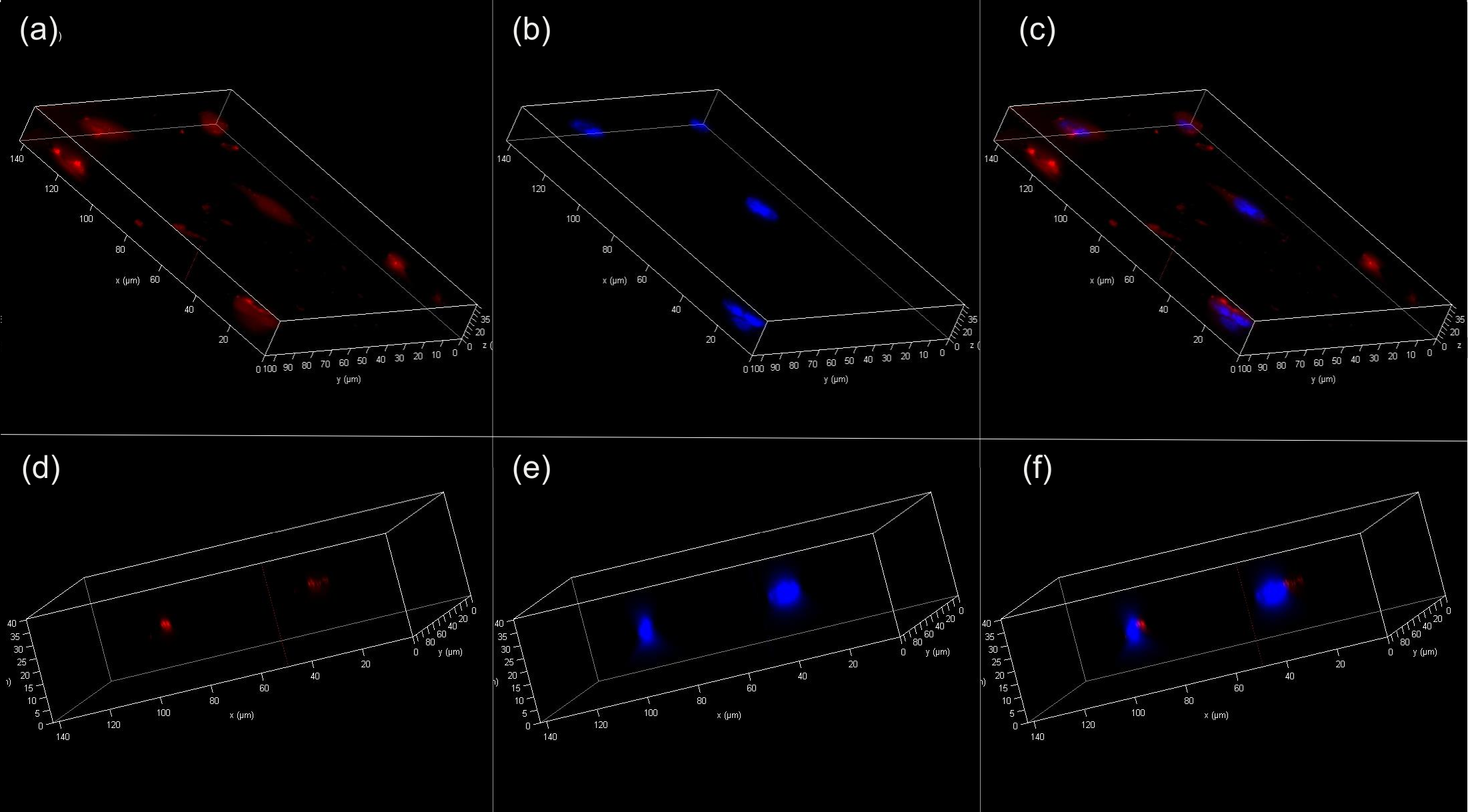
**Figure S7**: 3D reconstruction images of MDA-MB-231 cells after 8 h of incubation in the presence of (a-c) PSS/DOXO-coated GNRs@HSA/CS NPs and (d-e) (PSS-coated GNRs@HSA/CS NPs. (a, d) DOXO fluorescence is displayed in the red channel (λ_exc_ = 488 nm), and fluorescence from cell nuclei stained with DAPI is shown in the blue channel (λ_exc_ = 355 nm). (c,f) Merged images are also shown.

**S7. Cytotoxicity of bare HSA/CS and PSS-coated GNRs@HSA/CS NPs.**


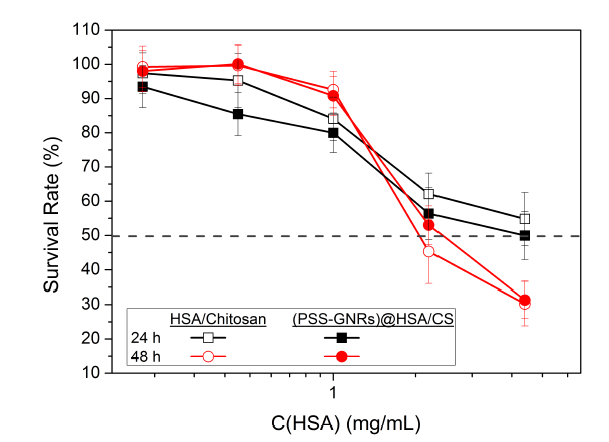


**Figure S8**: Cell viability of bare HSA/CS (open symbols) and PSS-coated GNRs@HSA/CS NPs (close symbols) in MDA-MB-231 breast cancer cells. HSA/CS and PSS-coated GNRs@HSA/CS NPs at 24h (□, ■) and 48 h (○, ●), respectively.

**S8. Chemotherapeutic effect of DOXO administered as free drug, and of PSS-coated GNRs@HSA/CS and PSS/DOXO-coated GNRs@HSA/CS NPs.**


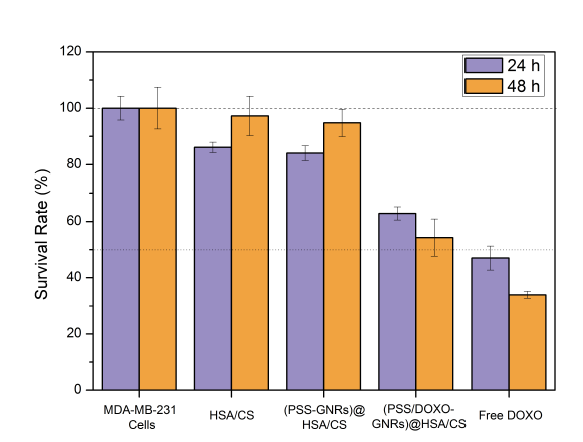


**Figure S9**: Survival rate (SR) of MDA-MB-231 breast cancer cells after 24 (blue) and 48 h (orange) of incubation in the presence of bare HSA/CS, PSS-coated GNRs@HSA/CS and PSS/DOXO-coated GNRs@HSA/CS NPs. Cells without any treatment were left as a negative control, and free DOXO was considered as the positive one.

**S.9** **Combination indices**

A synergistic effect is found when CI < 1 (log CI < 0), which means that the combined effect of both supplied drugs is greater than expected; an antagonism effect is observed when CI >1 (log CI >0), which implies the combinatorial effect is smaller than the additive effect; an additive effect is observed when CI = 1 (log CI= 0).


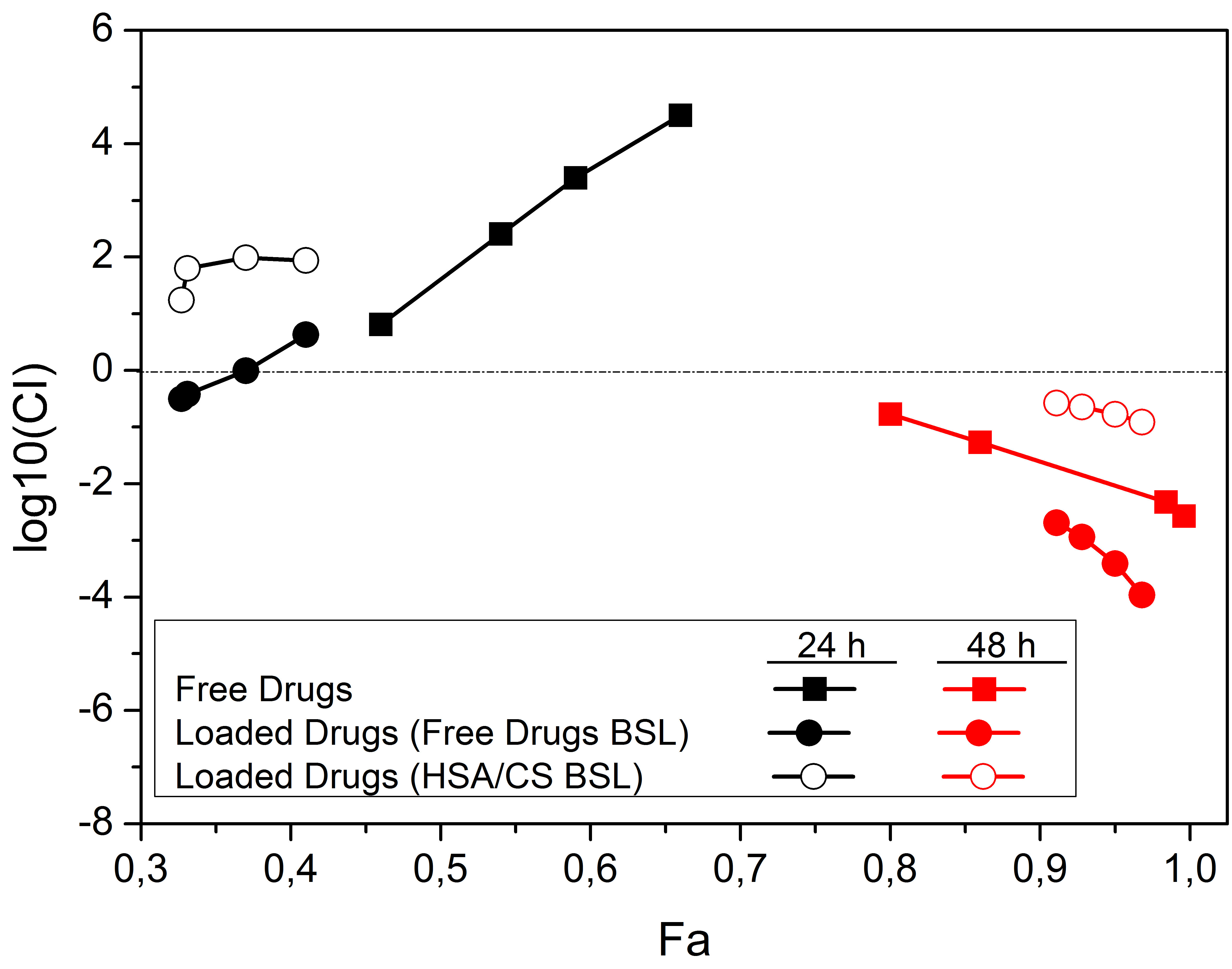


**Figure S10**: CIs for dual DTX and DOXO therapy using the hybrid HSA-based NPs at 24 h (black) and 48 h (red) of incubation. The evaluated formulations were the combination of both free drugs (DTX+DOXO) (■), and DTX+PSS/DOXOcoated-GNRs@HSA/CS hybrid NPs taking as baselines either the individual free drugs (○,○) or the encapsulated ones (●,●).

**Table S1**: CIs at 24 h and 48 h of incubation calculated with Compusyn software for dual-encapsulated and free DTX+DOXO, respectively. Calculations were made individually considering the free and encapsulated drugs as baselines.

| Free Drugs | | | |
| --- | --- | --- | --- |
| Doxo (µM) | **DTX (µM)** | **CI (24h)^*^** | **CI (48h)^*^** |
| 7.63 ± 0.81 | 3.67 ± 0.38 | 6.51 | 1.68·10^-1^ |
| 7.63 ± 0.81 | 14.89 ± 1.44 | 2.59·10^2^ | 5.40·10^-2^ |
| 7.63 ± 0.81 | 34.73 ± 4.17 | 2.50·10^3^ | 4.70·10^-3^ |
| 7.63 ± 0.81 | 50.13 ± 5.73 | 3.32·10^4^ | 2.68·10^-3^ |
| Dual encapsulated drugs within NPs compared to free drugs | | | |
| Doxo (µM) | **DTX (µM)** | **CI (24h)** | **CI (48h)** |
| 7.63 ± 0.81 | 3.67 ± 0.38 | 17.55 | 0.27 |
| 7.63 ± 0.81 | 14.89 ± 1.44 | 64.60 | 0.23 |
| 7.63 ± 0.81 | 34.73 ± 4.17 | 98.00 | 0.17 |
| 7.63 ± 0.81 | 50.13 ± 5.73 | 88.87 | 0.12 |
| Dual encapsulated drugs within NPs compared to encapsulated single ones | | | |
| Doxo (µM) | **DTX (µM)** | **CI (24h)** | **CI (48h)** |
| 7.63 ± 0.81 | 3.67 ± 0.38 | 0.32 | 2.02·10^-3^ |
| 7.63 ± 0.81 | 14.89 ± 1.44 | 0.38 | 1.15·10^-3^ |
| 7.63 ± 0.81 | 34.73 ± 4.17 | 0.98 | 3.91·10^-4^ |
| 7.63 ± 0.81 | 50.13 ± 5.73 | 4.26 | 1.08·10^-4^ |

^*Uncertainties of CI values are below 20%^

As additional information, the relative sensitivity (Z score) of a cell line to a given drug was derived. If Z is near 2, cell lines show resistance to this drug, whether Z is close to -2 the cell line is more sensitive to the chemo-effect. For MDA-MB-231 cells, Z values of 1.17 for DOXO and -0.17 for DTX were obtained. These would corroborate the high sensitivity of these cells to DTX derived from the *in vitro* cytotoxicity data and calculated IC_50_ values from Compusyn software.

**REFERENCES**

S1. Provencher SW, Stepanek P. Global analysis of dynamic light scattering autocorrelation functions. Part. Syst. Charact. 1996;13:291-294.

S2. Willets KA, Van Duyne RP. Localized surface plasmon resonance spectroscopy and sensing. Ann. Rev. Phys. Chem. 2007;58:267–297.

S3. Kekicheff P, Spalla O. Refractive index of thin aqueous films confined between two hydrophobic surfaces. Langmuir 1994;10:1584–1591.

S4. Huschka R, Zuloaga J, Knight MW, Brown LV, Nordlander P, Halas NJ. Light-induced release of DNA from gold nanoparticles: Nanoshells and nanorods. J. Am. Chem. Soc. 2011;133:12247–12255.

S5. Mulvaney P, Giersig M, Henglein A. Surface chemistry of colloidal gold: deposition of lead and accompanying optical effects. J. Phys. Chem., 1996;96:10419–10424.

S6. Dou J, Zhang H, Liu X, Zhang M, Zhai G. Preparation and evaluation in vitro and in vivo of docetaxel loaded mixed micelles for oral administration. Colloids Surfaces B: Biointerfaces, 2014;114:20–27.

S7. Upadhyay KK, Bhatt AN, Castro E, Mishra AK, Chuttani K, Dwarakanath BS, Schatz C, Le Meins JF, Misra A, Lecommandoux S. In vitro and in vivo evaluation of docetaxel loaded biodegradable polymersomes. Macromol. Biosci. 2010;10:503-512.

S8. Peralta DV, Heidari Z, Dash S, Tarr MA. Hybrid paclitaxel and gold nanorod-loaded human serum albumin nanoparticles for simultaneous chemotherapeutic and photothermal therapy on 4T1 breast cancer cells. ACS Appl. Mater. Interfaces. 2015;7: 7101–7111.
